# Repeated theta burst stimulation of the right ventrolateral prefrontal cortex reveals strong habituation in the context of stress and rumination

**DOI:** 10.1038/s41598-025-15099-1

**Published:** 2025-08-23

**Authors:** Isabell Int-Veen, Cosima Eisenlohr, Ramona Täglich, Betti Schopp, Hans-Christoph Nuerk, Christian Plewnia, Stefanie De Smet, Marie-Anne Vanderhasselt, Agnes Kroczek, Beatrix Barth, Andreas J. Fallgatter, Ann-Christine Ehlis, David Rosenbaum

**Affiliations:** 1 Tübingen Center for Mental Health (TüCMH), Department of Psychiatry and Psychotherapy, University Hospital Tübingen, Calwerstraße 14, 72076 Tübingen, Germany; 2 Department of Psychology, University of Tübingen, Tübingen, Germany; 3 German Center for Mental Health (DZPG), Partner Site Tübingen, Tübingen, Germany; 4 Department of Head and Skin, Psychiatry and Medical Psychology, Ghent University Hospital, Ghent University, Ghent, Belgium; 5 Ghent Experimental Psychiatry (GHEP) Lab, Ghent, Belgium; 6 Brain Stimulation and Cognition (BSC) Lab, Department of Cognitive Neuroscience, Faculty of Psychology and Neuroscience, Maastricht University, Maastricht, The Netherlands; 7 LEAD Graduate School and Research Network, University of Tübingen, Tübingen, Germany

**Keywords:** Theta Burst Stimulation, Stress, Rumination, Trier Social Stress Test, VLPFC, Appraisal, Psychology, Human behaviour

## Abstract

Prefrontal hypoactivation under stress has been consistently observed in individuals with high trait rumination and in patients with depression. However, no study has yet investigated the mechanisms of modulating activity of the Ventrolateral Prefrontal Cortex (VLPFC) by using excitatory and inhibitory Theta Burst Stimulation (TBS) in this context. To address this, we recruited 89 healthy participants (44 low trait ruminators and 45 high trait ruminators) who attended two lab sessions, each including stress induction via the Trier Social Stress Test (TSST). Participants received either excitatory intermittent TBS (iTBS) or inhibitory continuous TBS (cTBS). One session involved active TBS, while the other used sham TBS (sTBS), with the order randomized and balanced across stimulation conditions and groups. In high ruminators, we observed a significant impact of iTBS in the hypothesized direction when considering cortical oxygenation in the right VLPFC. We found higher subjective stress following iTBS and cTBS compared to sTBS during recovery of the TSST but only at the first appointment. No stimulation-dependent effects were found for other measures (state rumination, positive and negative affect, and heart rates). In our discussion, we draw conclusions concerning repeated-measures designs in TBS-studies.

**Supplementary Information:**

The online version contains supplementary material available at 10.1038/s41598-025-15099-1.

## Introduction

Non-Invasive Brain Stimulation (NIBS) comprises techniques to alter neural activation. One form of NIBS is Transcranial Magnetic Stimulation (TMS), which is capable of inducing excitability changes in the cortex and investigating the causal involvement of brain regions. TMS exists in several forms, such as single-pulse, paired-pulse, and repetitive TMS (rTMS), with rTMS shown to produce effects that last beyond the stimulation period^1^. A newer variant of rTMS, known as Theta Burst Stimulation (TBS), uses high-frequency TMS bursts mimicking the hippocampal theta-rhythms^2^. Depending on the interpulse and intertrain intervals, TBS can induce excitatory effects, thought to resemble long-term potentiation (intermittent TBS; iTBS), or inhibitory effects, akin to long-term depression (continuous TBS; cTBS)^3^.

TMS has been extensively investigated, particularly as a potential treatment for depression by modulating the left Dorsolateral Prefrontal Cortex (DLPFC)^4–6^. This is because prefrontal hypoactivation of the left DLPFC in patients with depression was observed during “affective and cognitive tasks requiring emotional or stress regulation, cognitive control, and/or shifting attention to external task demands”^7^. This means that, while increases in prefrontal brain activity—particularly in regions of the Fronto-Parietal Network (FPN) such as the DLPFC—are generally expected when moving from non-stressful to stressful tasks, individuals with depression consistently show attenuated increases in activation. This has been theorized to result at least in part from rumination, which is defined as “repetitive, prolonged, and recurrent negative thinking about one’s self, feelings, personal concerns and upsetting experiences”^8^. Recently, rTMS has been extensively investigated both as a treatment for depression—particularly via modulation of the left DLPFC—and in experimental contexts for its effects on rumination and the psychophysiological stress response^4–6,9–11^. While the left DLPFC is an important brain region involved in emotion regulation, cognitive control and the appropriate response to stressors, the Ventrolateral Prefrontal Cortex (VLPFC)—which is also part of the FPN—is proposed to be responsible for signaling the need to regulate to the DLPFC in the first place^12^. More specifically, the VLPFC initiates the appraisal and evaluates salience and indicates the need to regulate^12^. This is supported by reviews and meta-analyses of neuroimaging studies of cognitive reappraisal^13,14^. A review by Moses and colleagues^15^ highlights the importance of the VLPFC for studies using NIBS to investigate cognitive and emotional responses to psychosocial stressors, as “[...] the VLPFC—a key structure in cognitive reappraisal circuits—may be the best-supported target to affect stress modulation of emotional responses”.

Although research on the VLPFC remains limited compared to the left DLPFC, existing NIBS studies suggest that stimulation of the VLPFC improves emotion regulation and cognitive reappraisal^16–23^. This may especially be the case in social situations. Three studies combined the well-established Cyberball paradigm—a virtual ball-toss game where feelings of inclusion or exclusion are manipulated using the number of times participants receive the ball—with tDCS targeting the right VLPFC^24–26^ and showed that excitatory anodal tDCS applied to the right VLPFC buffers against social exclusion while the converse effects have been found for cathodal stimulation. Although the aforementioned studies did not include neural measures, their findings suggest that increased activation of the right VLPFC—also in more ecologically valid settings—may reduce emotional distress in socially challenging situations, such as social exclusion, likely by exerting top-down control over limbic regions. 

Beyond ostracism and social stress, early findings suggest the Cyberball paradigm also induces ruminative thinking^27,28^. Originally defined in the context of depression as persistent focus on symptoms and their implications^29^, rumination was considered a stable, situation-unspecific trait. Newer theories conceptualize it as a state-like process triggered by stress, observable even in healthy individuals^30–32^. Some researchers propose rumination functions as an emotion regulation strategy^33 ^and rather than being limited to depression, rumination has been shown to contribute to various mental disorders^34,35^. 

Using Cyberball and an emotion regulation task, Fowler et al.^27^ found that amygdala–VLPFC connectivity mediated the link between stress-reactive rumination and depressive symptoms in adolescent girls. Yet, the causal role of the VLPFC in adult stress-reactive rumination remains unclear.

Another widely used stress-induction is the Trier Social Stress Test (TSST)^36 ^which combines public speaking and mental arithmetic in front of an unresponsive panel. The TSST reliably induces stress and is considered the gold standard in human stress research^37,38^. It also elicits rumination in an ecologically valid way^39–47^ and can be used repeatedly^48–56 ^though longer intervals (10–16 weeks) are recommended to avoid habituation^51,56^. TSST-induced rumination has been linked to reduced prefrontal activity, especially in the DLPFC and Inferior Frontal Gyrus (IFG)^42,43,45,57^. So far, NIBS studies in the context of rumination have targeted only the left DLPFC. De Witte et al.^10^ observed interactions with trait rumination, which moderated TBS-effects on the physiological and psychological stress response. Era et al.^58^ observed elevated heart rate and cortisol, and lower HRV when cTBS was applied before a rumination induction. 

Using 20-Hz HF-rTMS applied to the left DLPFC after the TSST, Wang et al.^59^ observed reduced stress-induced cortisol increases. De Smet et al.^39^ applied cTBS, iTBS, or sTBS in a between-subjects design and found reduced cortisol responses in high ruminators following cTBS. Active TBS further enhanced heart rate recovery. Further assessing the neural correlates using functional near-infrared spectroscopy (fNIRS) and using a within-subject design^60^, no impact on heart rates but higher increases in negative affect and perceived stress were observed when high ruminators received iTBS. Overall, strong habituation effects in terms of most pronounced responses to the TSST in case of first-time exposure were observed.

These findings suggest that active TBS may have the potential to reduce rumination following the TSST and to modulate the stress response, highlighting their possible utility in mitigating stress-related cognitive and physiological processes.

In the current study, we aimed to investigate the causal role of the VLPFC. The present study offers a unique contribution to the field by employing a high-powered, sham-controlled, double-blind design to investigate the causal role of the right VLPFC in the stress-rumination-link. To our knowledge, this is the first study to apply both excitatory and inhibitory TBS to the right VLPFC prior to the TSST. Participants attended two laboratory sessions, receiving active stimulation in one session (iTBS or cTBS) and sham stimulation in the other, with the order of conditions randomized between subjects. Following the stimulation, participants underwent stress induction via the TSST. Our primary outcomes were stress, state rumination and negative affect. Moreover, we extended previous work by incorporating neural correlates of stimulation effects using fNIRS, thereby providing a more comprehensive understanding of the neurobiological mechanisms underlying stress-related rumination. Specifically, we assessed cortical oxygenation in our stimulation target, the VLPFC, which is known to play a key role in emotion regulation within social contexts. Additionally, we examined the DLPFC, given its involvement in emotion regulation, cognitive control, and adaptive responses to stressors. Finally, we measured the Somatosensory Association Cortex (SAC) as a proxy for the ventral Posterior Parietal Cortex—a critical component of the Central Executive Network (CEN)—since direct recording from this region was not feasible with our NIRS probe configuration. The SAC was selected as it constitutes the closest accessible cortical area. 

We hypothesized that modulation of the VLPFC via TBS would influence prefrontal activation under stress and, in turn, affect emotion regulation such as cognitive reappraisal and state rumination. Specifically, we expected that activation of the VLPFC would facilitate adaptive reappraisal and thereby suppress state rumination. Accordingly, we predicted that inhibitory cTBS of the VLPFC would lead to reduced prefrontal activation under stress, particularly in high ruminators, exacerbating their pre-existing prefrontal dysfunction. In contrast, excitatory iTBS should increase VLPFC-activation, thereby potentially normalizing prefrontal functioning in high ruminators. For low ruminators, we expected a smaller impact of TBS due to their already high prefrontal engagement under stress. Specifically, cTBS was expected to reduce VLPFC-activation, whereas iTBS might lead to slightly increased cortical oxygenation. 

Consequently, we hypothesized that iTBS would result in reduced stress, negative affect, and state rumination. Conversely, cTBS was expected to impair emotion regulation, leading to increased stress, negative affect, and rumination, particularly in high ruminators.

## Results

### Sample

Overall, the mean age of the sample was 23.82 years (*SD* = 6.00), and a total of 68.54% of the sample were female. Participants scored a mean RRS of 2.01 (*SD* = 0.64) and a BDI-II of 6.07 (*SD* = 5.99) equaling “no depression”^61^. There were no differences concerning demographic variables among the two study arms (Table 1).

**Table 1:** Demographic variables of the sample by study arm.

	cTBS-arm	iTBS-arm	Test statistic	Total sample
Age	23.45 (6.15)	24.18 (5.89)	*F*(1,87) = 0.321, *p* = .572, *η*_*p*_^*2*^ = 0.004	23.82 (6.00)
Percent female	72.7%	64.4%	$\:{\chi\:}^{2}$(1) = 0.376, *p* = .540	68.54%
BDI-II total score	6.36 (5.73)	6.38 (6.21)	*F*(1,87) = 0.00, *p =* .991, *η*_*p*_^*2*^ = 0.000	6.07 (5.99)
RRS mean	2.06 (0.62)	1.89 (0.55)	*F*(1,87) = 1.97, *p* = .164, *η*_*p*_^*2*^ *=* 0.02	2.01 (0.64)

*BDI-II* = Beck Depression Inventory II, *RRS =* Ruminative Response Scale. Test statistic = comparison of the cTBS- and iTBS-arm. Please note that the RRS and BDI-II scores correspond to assessments at the first appointment.

### Blinding and comparability of motor thresholds

A binomial test testing whether participants correctly identified whether they received active or sham stimulation (true vs. false; *H*_0_: *p* = .5) showed that participants could not distinguish between sham and active stimulation during the first, but blinding failed at the second appointment (supplemental material S1). Note that while we did not specifically recruit participants with no prior experience with TMS/tDCS, only 5 had experienced TMS, 3 of whom had also used tDCS, and 5 had experienced tDCS but not TMS. There were no differences in motor thresholds between cTBS (*M* = 43.89, *SD* = 6.93), iTBS (*M* = 40.80, *SD* = 5.20) and sTBS (*M* = 42.18, *SD* = 6.06), *F*(2, 175) = 2.872, *p =* .059, *η*_*p*_^*2*^ = 0.03.

### Subjective stress: contrasts

**Fig. 1:**
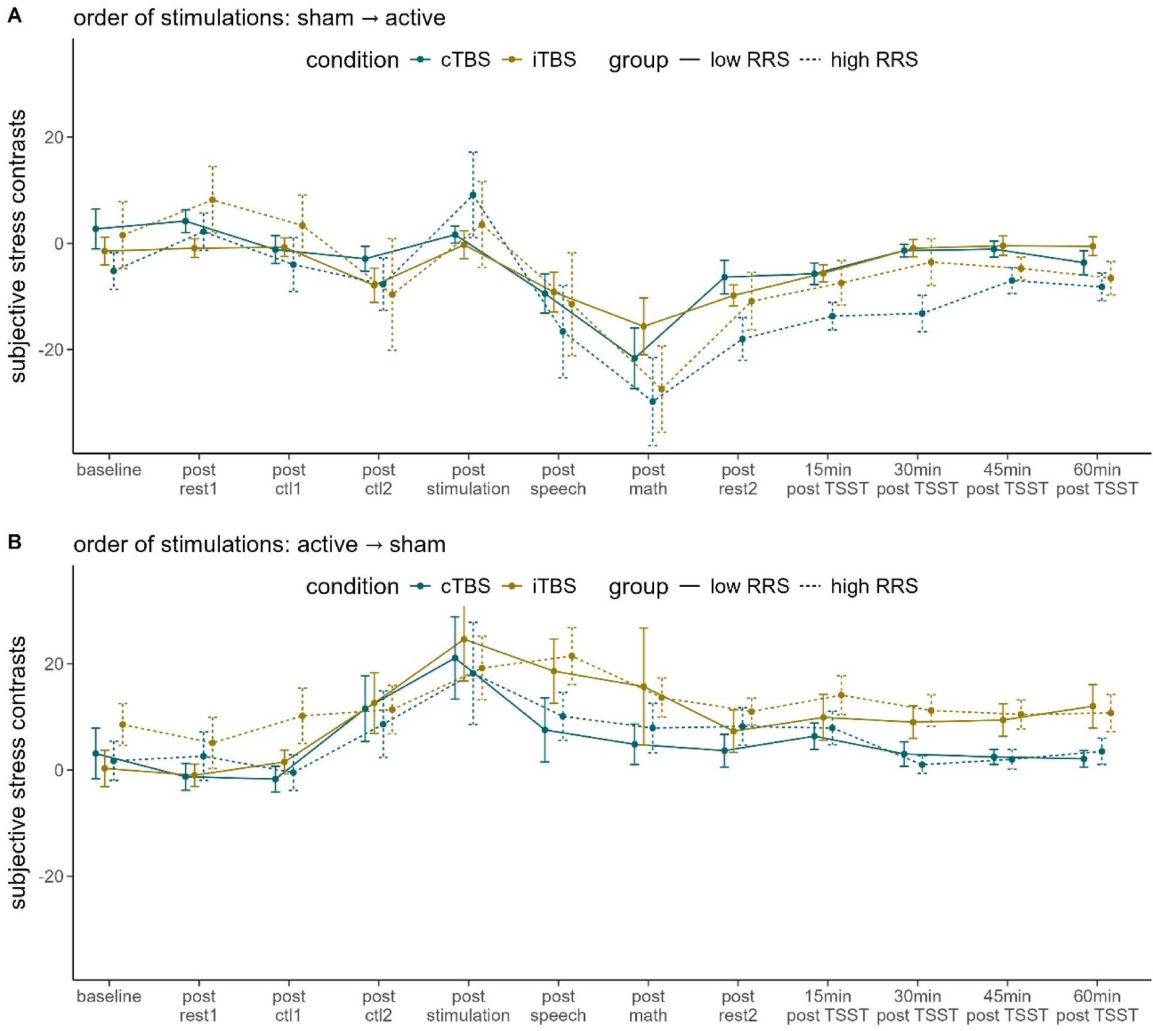
Line plot of the contrasts of subjective stress ratings dependent on order of conditions (**A**  sham stimulation at the first appointment, active stimulation at the second appointment; **B**  active stimulation at the first appointment, sham stimulation at the second appointment). *rest* = resting-state measurement, *ctl1 =* control task 1, *ctl2 = *control task 2, *speech =* job interview of the TSST, *math =* arithmetic task of the TSST, *TSST =* Trier Social Stress Test, *sTBS =* sham Theta Burst Stimulation, *cTBS* = continuous Theta Burst Stimulation, *iTBS =* intermittent Theta Burst Stimulation, *15 min * = 15 min after the TSST, *RRS* = Ruminative Response Scale. Error bars indicate 1 standard error of the mean.

We first fitted a rmANOVA for stress contrasts (active minus sham) as a function of the four-way interaction of time (indicating the 12 repeated assessments of stress over the course of one appointment), RRS-group (low vs. high-ruminators), stimulation condition (cTBS vs. iTBS) and order of stimulation conditions (active → sham vs. sham → active). As a result, we observed a significant interaction of time and order of stimulation conditions, *F*(5.617, 432.473) = 14.037, *p < .*001, $\:{\eta\:}_{p}^{2}$ = 0.154, which is why we then fitted separate rmANOVAs dependent on the order of stimulation conditions. In both cases, we only observed a significant main effect of time (active → sham: *F*(4.595, 174.603) = 7.703, *p < .*001, $\:{\eta\:}_{p}^{2}$ = 0.169; sham → active: *F*(5.059, 197.320) = 16.269, *p < .*001, $\:{\eta\:}_{p}^{2}$ = 0.294) and a main effect of the constant term (i.e. a significant main effect of active stimulation vs. sham) [active → sham: *F*(1, 38) = 40.338, *p < .*001, $\:{\eta\:}_{p}^{2}$ = 0.515 (Fig. 1A); sham → active: *F*(1, 39) = 14.732, *p < .*001, $\:{\eta\:}_{p}^{2}$ = 0.274 (Fig. 1B)]. To evaluate the time course in greater detail, we investigated polynomial contrasts, which revealed a significant quadratic time course illustrating the stress increase due to the TSST and subsequent decrease in the post-stress phase. This effect, however, was inversely dependent on the order of stimulation conditions. When participants received sTBS first, stress ratings were U-shaped, *F*(1, 39) = 19.636, *p < .*001, $\:{\eta\:}_{p}^{2}$ = 0.335; when participants received active first, stress was inversely U-shaped, *F*(1, 38) = 33.196, *p < .*001, $\:{\eta\:}_{p}^{2}$ = 0.466. This means that stress increases were higher in case of first-time exposure to the TSST.

Lastly, only when participants received active stimulation first, we observed a significant main effect of the stimulation, *F*(1, 38) = 4.443, *p < .*05, $\:{\eta\:}_{p}^{2}$ = 0.105, which was illustrated by increased differences between active and sham stimulation following iTBS compared to cTBS. This means that participants rated their stress overall higher when they received iTBS at the first appointment compared to when they received sTBS at the second appointment, whereas stress was more comparable between the two appointments when participants received cTBS. 

### Subjective stress: planned contrasts

**Fig. 2:**
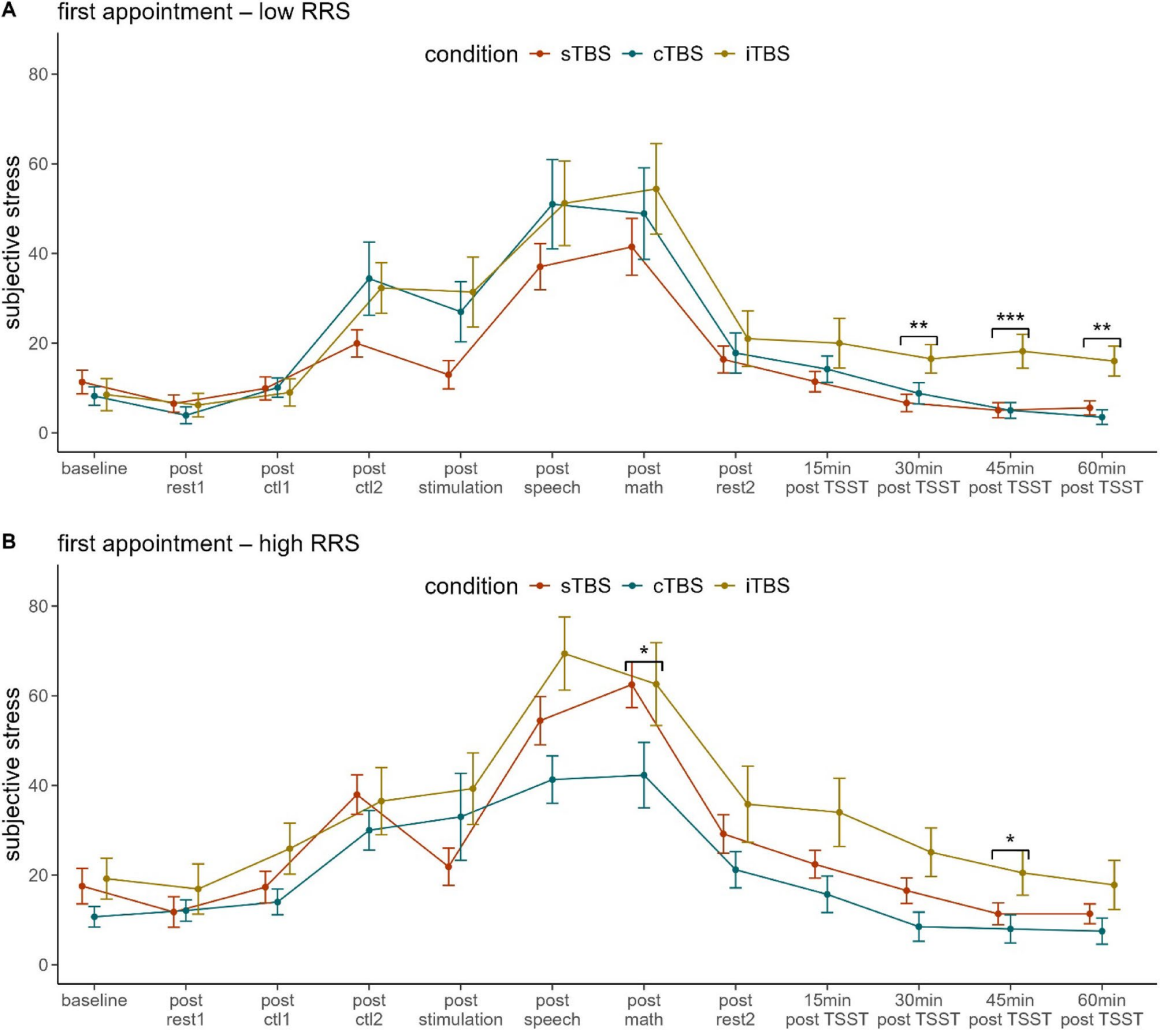
Illustration of the planned contrasts of subjective stress for low ruminators (**A**) and high ruminators (**B**). At the first appointment, we observed significantly higher subjective stress following iTBS compared to sTBS. In low ruminators, this effect was evident at 30 min (*p* < .01), 45 min (*p* < .001), and 60 min post TSST (*p* < .01). In high ruminators, significantly higher stress following iTBS compared to sTBS was observed at 45 min post TSST (*p* < .05). Additionally, when comparing cTBS and sTBS, high ruminators reported significantly higher subjective stress following sTBS compared to cTBS immediately after the TSST (*p* < .05). *rest* = resting-state measurement, *ctl1 =* control task 1, *ctl2 =* control task 2, *speech =* job interview of the TSST, *math* = arithmetic task of the TSST, *TSST =* Trier Social Stress Test, *sTBS =* sham Theta Burst Stimulation, *cTBS =* continuous Theta Burst Stimulation, *iTBS =* intermittent Theta Burst Stimulation, *15 min =* 15 min after the TSST, *RRS =* Ruminative Response Scale. Error bars indicate 1 standard error of the mean.

As planned contrasts, we compared stress between the iTBS and cTBS vs. sTBS at the six time points following the TSST. These comparisons were conducted separately for low and high ruminators across the different appointments. We observed higher stress following iTBS compared to sTBS, but only at the first appointment: Namely, we observed higher stress following iTBS compared to sTBS in low ruminators at 30 min (*M*_*sTBS *_= 7.27, *SD*_*sTBS*_ = 9.14; *M*_*iTBS *_= 16.50, *SD*_*iTBS*_ = 10.01, *t*(30) = –2.571, *p < .*01, *d *= –0.981), 45 min (*M*_*sTBS*_ = 5.73, *SD*_*sTBS*_ = 8.10; *M*_*iTBS *_= 18.20, *SD*_*iTBS *_= 12.01, *t*(30) = –3.463, *p < .*001, *d* = –1.321) and 60 min post TSST (*M*_*sTBS *_= 6.68, *SD*_*sTBS *_= 8.71; *M*_*iTBS*_ = 16.00, *SD*_*iTBS *_= 10.49, *t*(30) = –2.633, *p < .*01, *d* = -1.004) (Fig. 2A) and in high ruminators at 45 min post TSST (*M*_*sTBS *_= 11.36, *SD*_*sTBS*_ = 11.56; *M*_*iTBS *_= 20.50, *SD*_*iTBS *_= 15.71, *t*(30) = –1.851, *p *< .05, *d* = –0.703) (Fig. 2B).

When comparing cTBS and sTBS, we observed higher stress in high ruminators at the first appointment following sTBS (*M* = 62.50, *SD* = 24.25) compared to cTBS (*M* = 42.30, *SD* = 23.13) directly after the TSST, *t*(30) = 2.214, *p < .*05, *d* = 0.845 (Fig. 2B). 

### State rumination: contrasts

**Fig. 3:**
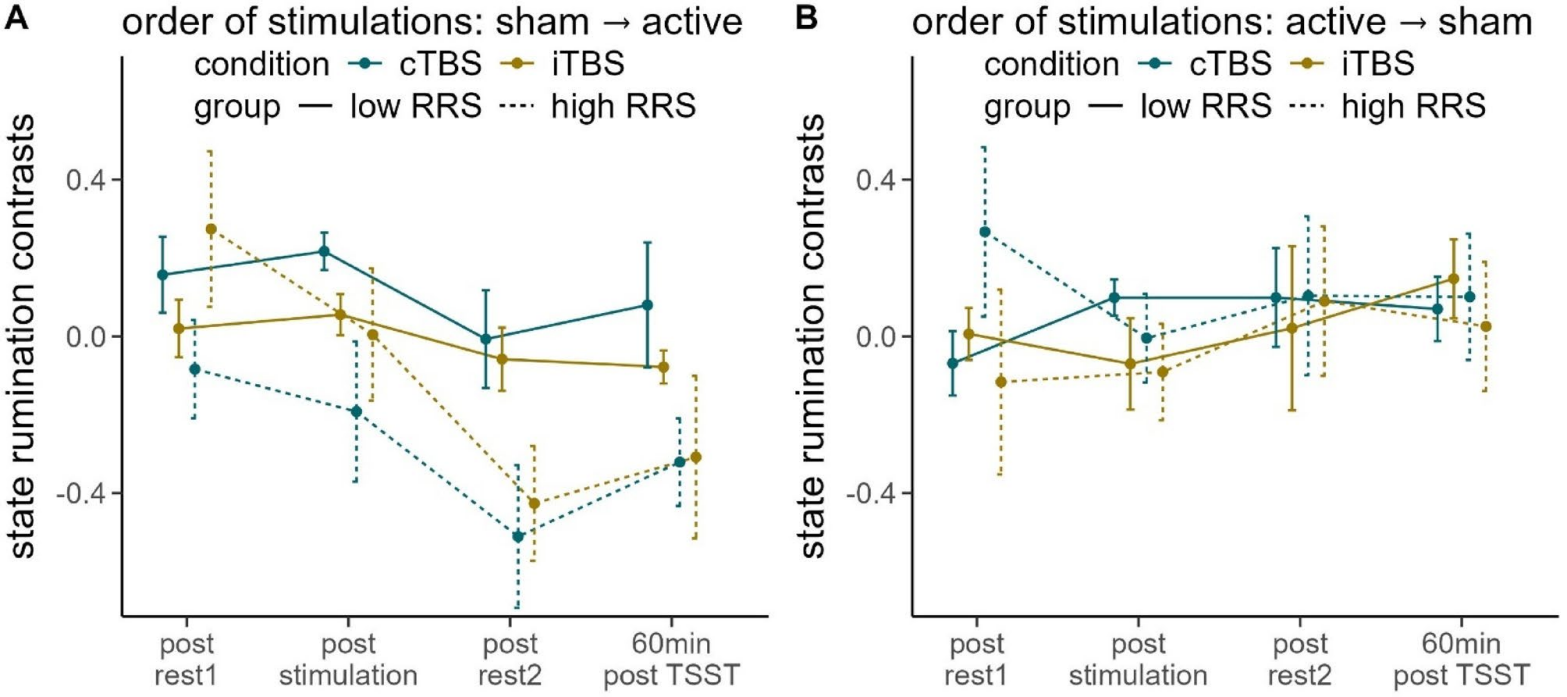
Line plot of the contrasts of state rumination ratings dependent on order of conditions (**A** sham stimulation at the first appointment, active stimulation at the second appointment;  **B** active stimulation at the first appointment, sham stimulation at the second appointment). *rest =* resting-state measurement, *TSST =* Trier Social Stress Test, *RRS* = Ruminative Response Scale. Error bars indicate 1 standard error of the mean.

Fitting a rmANOVA for state rumination contrasts (active minus sham) as a function of the four-way interaction of time (indicating the 4 repeated assessments of state rumination over the course of one appointment), RRS-group (low vs. high ruminators), stimulation condition (cTBS vs. iTBS) and order of stimulation conditions (active → sham vs. sham → active), we observed a significant interaction of time and order of stimulation conditions, *F*(2.917, 224.632) = 5.251, *p < .*01, $\:{\eta\:}_{p}^{2}$ = 0.064. We then fitted separate rmANOVAs dependent on the order of stimulation conditions. For participants having received sham stimulation first, we observed a significant main effect of time, *F*(3, 114) = 6.975, *p *< .001, $\:{\eta\:}_{p}^{2}$ = 0.155 and a significant main effect of RRS-group, *F*(1, 38) = 7.583, *p* < .01, $\:{\eta\:}_{p}^{2}$ = 0.166 (Fig. 3A).

Polynomial contrasts of the main effect of time revealed a significant linear contrast, *F*(1, 38) = 12.273, *p* < .01, $\:{\eta\:}_{p}^{2}$ = 0.244. This is reflected in comparable state rumination between active and sham stimulation prior to the stimulation and TSST, with increasing differences observed afterward. Following the TSST, we found negative contrasts, indicating higher state rumination following sham (i.e. their first appointment).

The main effect of group was reflected by negative contrasts: High ruminators experienced higher state rumination following sham compared to active stimulation (i.e. their first appointment), whereas low ruminators experienced lower rumination regardless of the stimulation.

No effects were observed when participants received active stimulation first (all *p*’s > 0.396) (Fig. 3B).

### State rumination: planned contrasts

When we investigated state rumination after the second resting-state separately for low and high ruminators on each appointment, we observed no differences (all *p’s* > 0.056).

### State rumination: reliable change

Lastly, we calculated Reliable Change Indices (RCI) using state rumination prior to the stress induction (rest1) to after the stress induction (rest2) for each appointment and RRS-group separately. In case of both appointments, most participants exhibited no reliable change in state rumination (Table 2). Interestingly, about 35% of high ruminators showed reliable increases at the first and only 13% at the second appointment. The same pattern was observed for low ruminators. Further, no low ruminator and only 3 high ruminators showed significant decreases at the first appointment; this was the case in 1 low and 12 high ruminators at the second appointment (see supplementary material S2).

**Table 2:** Absolute and relative frequencies of reliable change.

	Low ruminators (*n* = 44)	High ruminators (*n* = 45)	Test statistic
*Appointment 1:*			$\:{\chi\:}^{2}$(2) = 8.432, *p < .*05
Reliable decrease	0 (0.00%)	3 (6.67%)	
No reliable change	37 (84.09%)	26 (57.78%)	
Reliable increase	7 (15.91%)	16 (35.55%)	
*Appointment 2:*			$\:{\chi\:}^{2}$(2) = 14.181, *p < .*001
Reliable decrease	1 (2.27%)	12 (26.67%)	
No reliable change	41 (93.18%)	27 (60.00%)	
Reliable increase	2 (4.55%)	6 (13.33%)	

Percentages refer to the relative frequencies in the corresponding subsample (low or high ruminators) and the test-statistic indicated $\:{\chi\:}^{2}$-tests comparing the distribution of RCI-categories in low and high ruminators at the respective appointment.

### Negative affect: contrasts

**Fig. 4:**
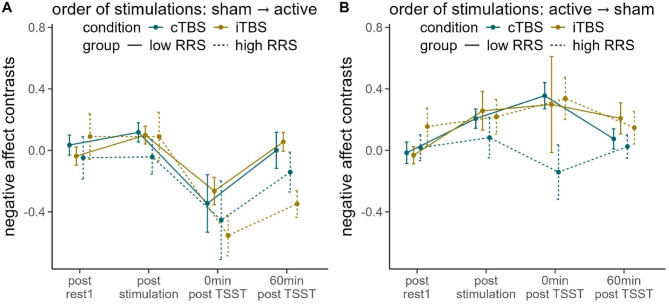
Line plot of the contrasts of negative affect ratings dependent on order of conditions (**A** sham stimulation at the first appointment, active stimulation at the second appointment; **B** active stimulation at the first appointment, sham stimulation at the second appointment). *rest =* resting-state measurement, *TSST* = Trier Social Stress Test, *RRS* = Ruminative Response Scale. Error bars indicate 1 standard error of the mean.

We conducted a rmANOVA on negative affect contrasts (active minus sham), with time (reflecting the 4 repeated assessments of the PANAS within one appointment), RRS-group (low vs. high ruminators), stimulation condition (cTBS vs. iTBS), and order of stimulation conditions (active → sham vs. sham → active) as factors. We observed a significant interaction of time and order of stimulation conditions, *F*(2.503, 192.699) = 9.012, *p* < .001, $\:{\eta\:}_{p}^{2}$ = 0.105. Fitting rmANOVAs dependent on the order of stimulation conditions, we observed a significant main effect of the constant term (i.e. a significant main effect of active stimulation vs. sham: sham → active: *F*(1, 39) = 13.297, *p* < .001, $\:{\eta\:}_{p}^{2}$ = 0.254) (Fig. 4A); active → sham: *F*(1, 38) = 8.505, *p* < .01, $\:{\eta\:}_{p}^{2}$ = 0.183 (Fig. 4B). Only in participants having received sham first, we observed a main effect of time, *F*(2.713, 103.079) = 11.566, *p* < .001, $\:{\eta\:}_{p}^{2}$ = 0.233. Investigating the main effect of time using polynomial contrasts, we observed a linear contrast, *F*(1, 38) = 8.781, *p* <.01, $\:{\eta\:}_{p}^{2}$ = 0.188, which was reflected by comparable negative affect between active and sham stimulation previous to the TSST and negative contrasts (higher negative affect following sTBS, i.e. the first appointment) afterwards.

### Negative affect: planned contrasts

We observed no significant differences in negative affect directly after the TSST (all *p*’s > 0.063).

### Cortical oxygenation: overall *t*-tests

**Fig. 5:**
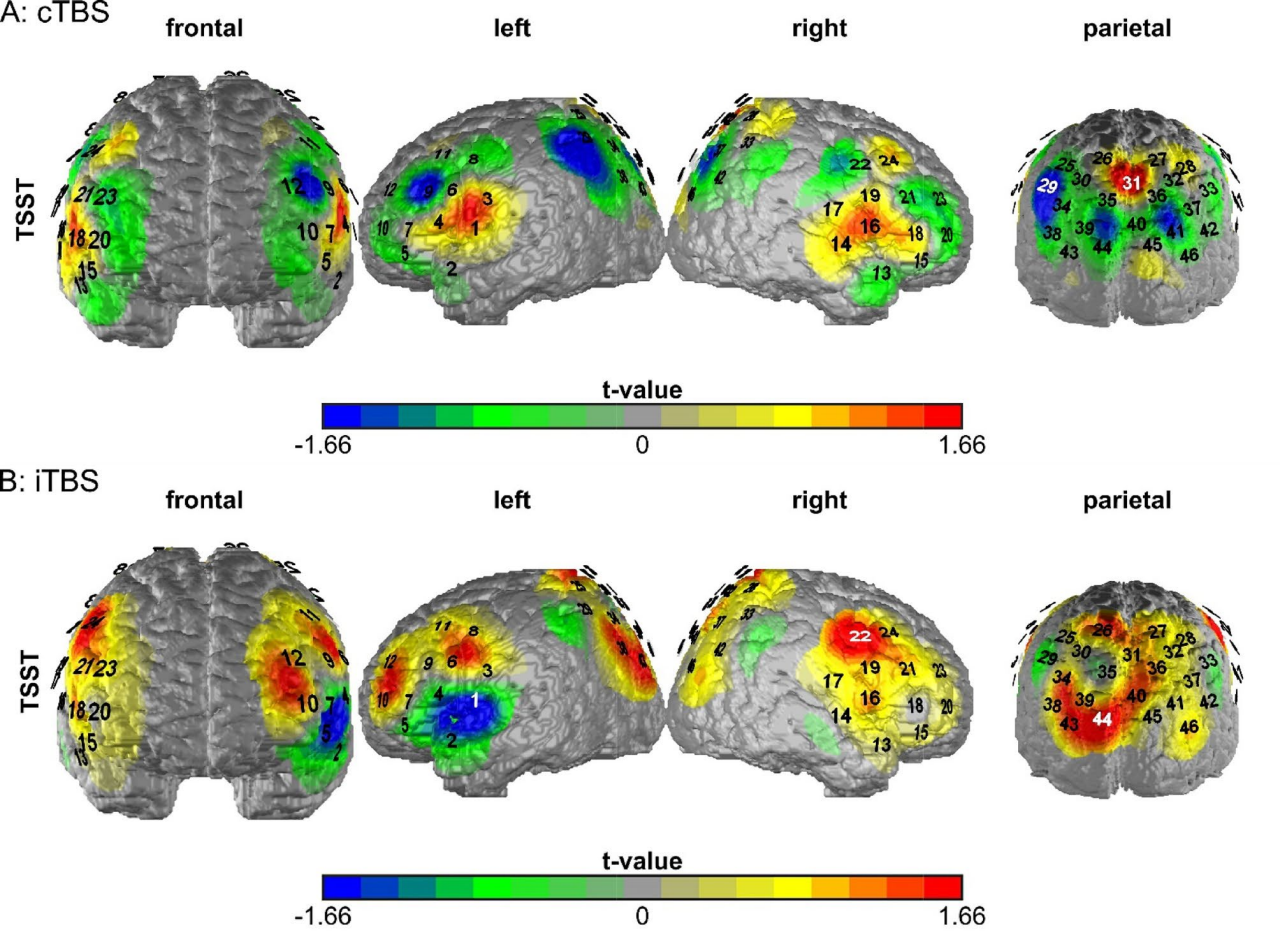
Plots of the *t*-values of the paired *t*-tests in each channel testing the active stimulation condition against the sham stimulation in each channel dependent on the stimulation condition. A = cTBS, B = iTBS, TSST = arithmetic task of the TSST. The figure legend was scaled so that channels showing a significant *t*-value are displayed in blue ($\:\:\le\:$ – 1.66) or red ($\:\:\ge\:$ 1.66). Significant channels are displayed in white to make them more easily identifiable. Significant channels in the cTBS condition are channel 29 and 31. Significant channels in the iTBS condition are channel 1, 22 and 44. This plot was generated using custom MATLAB 2024a scripts. Please note that, although we expected a negative difference after cTBS and a positive difference after iTBS, the results showed the opposite pattern in channel 31 (cTBS) and channel 1 (iTBS), respectively.

Paired *t*-tests comparing cortical oxygenation during the arithmetic task of the TSST between active and sham stimulation revealed significant effects in two channels for cTBS (channel 29 and 31; Brodmann area 7) (Fig. 5A), and three in the iTBS condition (channel 1, 22; both Brodmann area 6 and 44; Brodmann area 7) (Fig. 5B). Please note that, although we expected a negative difference after cTBS and a positive difference after iTBS, the results showed the opposite pattern in channel 31 (cTBS) and channel 1 (iTBS), respectively.

### Cortical oxygenation: contrasts single channel

We then fitted a rmANOVA with the four-way-interaction of time (reflecting the three tasks during which cortical oxygenation was assessed: control task 1, control task 2, and the arithmetic task of the TSST), RRS-group (low vs. high ruminators), stimulation condition (cTBS vs. iTBS), and order of stimulation conditions (active → sham vs. sham → active) to investigate whether stimulation had a significant effect in the single stimulated channel corresponding to electrode position F8. No significant effects were observed (all *p’s* > 0.085).

### Cortical oxygenation: contrasts ROIs

Lastly, we fitted a rmMANOVA using our 5 ROIs (bilateral DLPFC, bilateral VLPFC and SAC) dependent on the four-way interaction of time (reflecting the three tasks during which cortical oxygenation was assessed: control task 1, control task 2, and the arithmetic task of the TSST), RRS-group (low vs. high ruminators), stimulation condition (cTBS vs. iTBS) and order of stimulation conditions (active → sham vs. sham → active). We observed a significant interaction of time, RRS-group and stimulation condition, *F*(10, 310) = 2.097 *p* < .05, $\:{\eta\:}_{p}^{2}$ = 0.063. Univariate tests did not yield significance in any ROI.

**Fig. 6:**
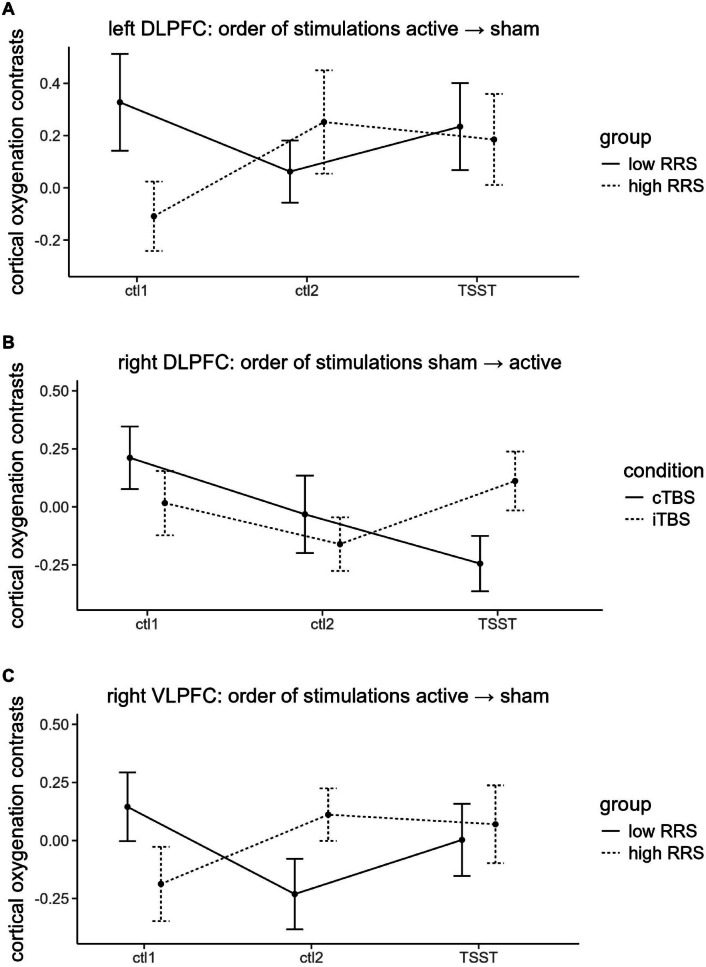
Illustration of the polynomial contrasts of the fNIRS analysis: (**A**) contrasts in the left DLPFC in case participants received active stimulation first; (**B**) contrasts in the right DLPFC in case participants received sham stimulation first; (**C**) contrasts in the right VLPFC in case participants received active stimulation first. *ctl1 =* control task 1 (reading numbers), *ctl2* = control task 2 (mental arithmetic without social stress), *TSST =* arithmetic task of the Trier Social Stress Test (mental arithmetic with social stress). Error bars indicate 1 standard error of the mean.

When investigating polynomial contrasts, we observed a significant linear contrast of the four-way interaction of time, RRS-group, stimulation condition and order of stimulation conditions in the left DLPFC, *F*(1, 79) = 4.250, *p* < .05, $\:{\eta\:}_{p}^{2}$ = 0.051. This seemed to be driven by group differences previous to the TBS, namely positive contrasts in the low ruminators compared to negative contrasts in the high ruminators during control task 1 but only in case participants received active stimulation first (Fig. 6A).

We further observed a significant linear contrast of the three-way interaction of time, condition and order of stimulation condition in the right DLPFC, *F*(1, 79) = 4.607, *p < .*05, $\:{\eta\:}_{p}^{2}$ = 0.055. This was primarily driven by participants having received sham stimulation at their first and active stimulation at the second appointment: During control task 1, participants having received iTBS and participants having received cTBS exhibited comparable, positive contrasts (higher cortical oxygenation at the second appointment, i.e. during active stimulation). During control task 2, participants having received iTBS and cTBS showed comparable, negative contrasts (higher cortical oxygenation at the first appointment, i.e. during sham stimulation). During the arithmetic task of the TSST, this pattern was reversed: Participants having received iTBS showed positive contrasts (higher cortical oxygenation at the second appointment, i.e. during active stimulation), while participants having received cTBS showed negative contrasts (higher cortical oxygenation at the first appointment, i.e. during sham stimulation) (Fig. 6B). 

We also observed a quadratic contrast of the three-way interaction of time, group and order of stimulation conditions in the right VLPFC, *F*(1, 79) = 5.420, *p* < .05, $\:{\eta\:}_{p}^{2}$ = 0.064. This was reflected by similar contrasts between low and high ruminators at every given time point and quasi no changes over time in case participants received sham stimulation first. In case they received active stimulation first, low ruminators exhibited positive contrasts (higher cortical oxygenation at the first appointment, i.e. during active stimulation) while high ruminators exhibited negative contrasts (higher cortical oxygenation at the second appointment, i.e. during sham stimulation) during control task 1. During control task 2, this pattern was reversed and during the arithmetic task, both groups exhibited comparable positive contrasts (for brainmaps see supplementary material S3) (Fig. 6C). We observed no significant between-subjects effects.

### Cortical oxygenation: planned contrasts

**Fig. 7:**
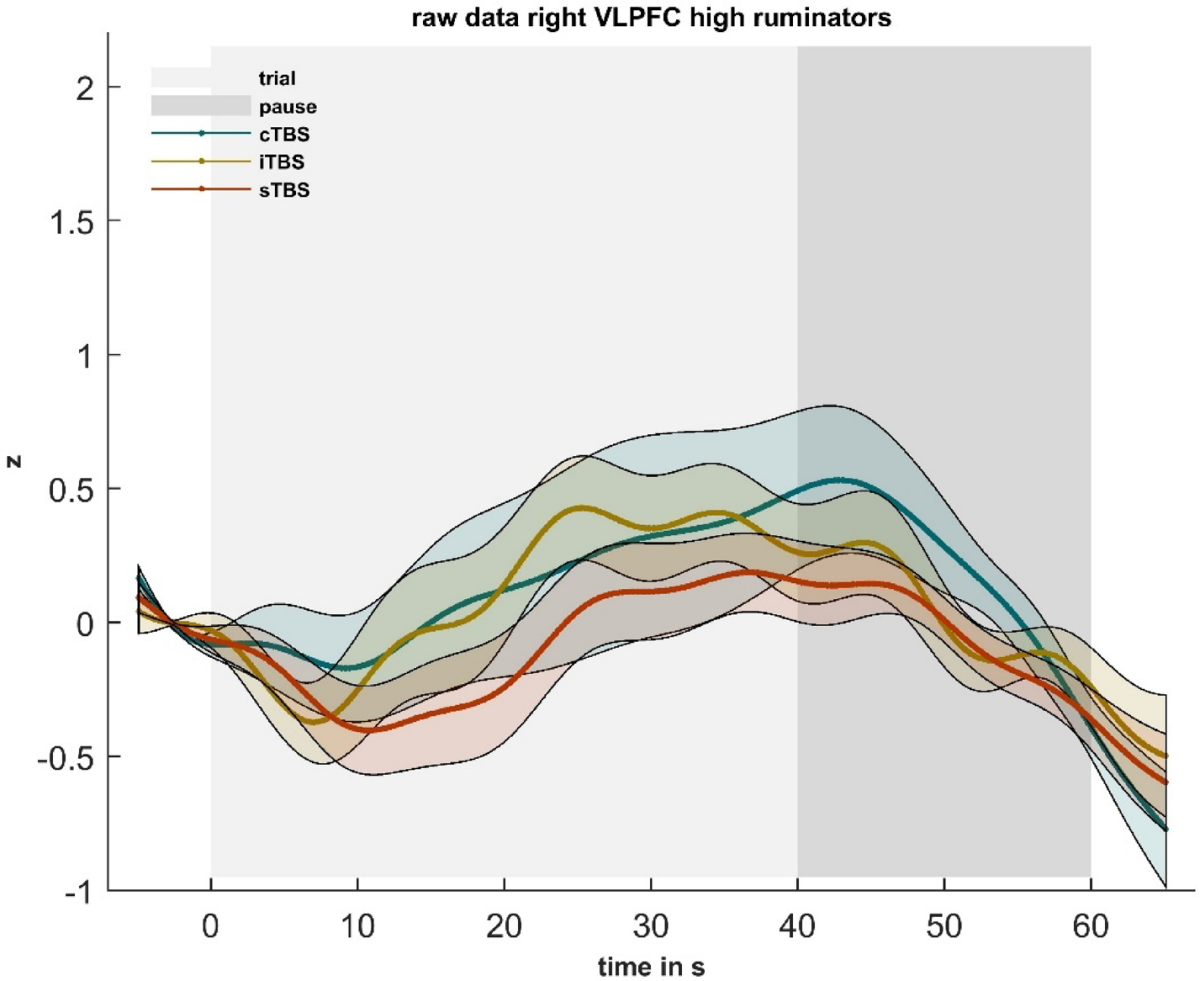
Illustration of the z-standardized hemodynamic responses during the arithmetic task of the TSST in the right VLPFC in high ruminators depending on the TBS-condition at the first appointment. The light shading marks the 40 s trial and the dark shading the 20 s pause to allow the hemodynamic response to recover. Shadings around the hemodynamic curves reflect standard errors of the mean. The baseline includes the 5 s before each trial; 0 s on the x-axis marks the beginning of the trial. See supplementary material S17 for the time series of the other ROIs.

Investigating our planned contrasts, we observed significantly higher cortical oxygenation in the right VLPFC during the arithmetic task following iTBS for high ruminators at the first appointment (*M* = 0.83, *SD =* 0.51) compared to sTBS (*M* = 0.40, *SD* = 0.51), *t*(32) = –2.268, *p* < .05, *d* = –0.831 (Fig. 7).

### Impact of expectancy effects

We observed a main effect of expectancy effects during the first appointment, *F*(2, 59) = 5.659, *p* < .01, $\:{\eta\:}_{p}^{2}$ = 0.161, and the second appointment for stress, *F*(2, 62) = 3.212, *p* <.05, $\:{\eta\:}_{p}^{2}$ = 0.094. We observed a significant interaction of time and expectancy effects during the first appointment for negative affect, *F*(1.972, 138.055) = 3.921, *p* <.05, $\:{\eta\:}_{p}^{2}$ = 0.053. No significant expectancy effects emerged when included in the fNIRS-analysis.

## Discussion

The study at hand aimed to investigate the effects of TBS applied to the right VLPFC on the physiological and behavioral stress response. In general, replicating previous findings^37,62 ^we observed stronger responses to the TSST in terms of stress, state rumination, and negative affect during first-time exposure to the paradigm. This is most likely due to a reduced feeling of uncontrollability as the experimental stress induction was very similar for both appointments despite different starting points, different calculations and a change in TSST jury members. Nevertheless, the stress induction was also successful in the case of the second exposure to the TSST. 

For stress, we observed a significant main effect of the stimulation but only in participants receiving active stimulation first: Participants rated their stress overall higher when they received iTBS at the first appointment compared to when they received sTBS at the second appointment. In contrast, stress was more consistent and lower across both appointments when participants received cTBS. When we investigated the post-stress phase, we found higher stress in low and high ruminators having received iTBS compared to participants having received sTBS but only at the first appointment. This is contrary to our initial hypothesis where we expected a positive effect on stress levels due to the suggested excitatory effect on the right VLPFC and therefore facilitation of adaptive emotion regulation. Potentially, the uncomfortableness of the stimulation may have led to higher stress that may be experienced as more intense compared to a smaller effect of facilitated emotion regulation. Further, the rating of stress using a Visual Analogue Scale might not be the optimal form of assessment because several aspects of rating how stressful an experience is are merged in a single number which makes it impossible to evaluate why participants rated their stress the way they did. It is also important to note that many studies investigating the VLPFC so far were conducted using tDCS^16–18 ^which is often perceived as less painful. Especially due to the relative closeness to facial nerves and muscles, future investigations using TBS applied to the VLPFC are desperately needed to disentangle the potentially opposing effects of experienced discomfort and the facilitation of emotion regulation on stress.

With respect to state rumination, our results showed no clear effect of the stimulation conditions, but again an effect of order of stimulation conditions, reflecting habituation effects between the TSST-sessions. Moreover, high ruminators exhibited significantly higher state rumination compared to low ruminators, consistent with prior research^43–45^. 

As reflected by reliable change indices, low ruminators showed low or no increases in state rumination while strong increases were found in the high ruminators at the first appointment. 

We further observed higher negative affect in high-ruminators and more pronounced increases in case of the first-time exposure, however, no impact of the stimulation. 

Considering performance measures of the TSST, we again observed no significant impact of the stimulation but of the order of stimulation conditions. Also, heart rates indicated successfully induced stress at both appointments but no differences among stimulation conditions. Please note that analysis of performance measures and heart rates are to be found in the supplementary material. 

The absent effect of TBS might be explained by several factors, e.g. the timing of the stimulation and time passing between the TBS and the TSST or potentially suboptimal targeting of the right VLPFC. Indeed, the VLPFC is difficult to target due to the closeness to facial nerves and muscles. However, we observed hypothesis-conform differences in cortical oxygenation in the right VLPFC during the TSST in high ruminators: At the first appointment, we observed higher cortical oxygenation following iTBS compared to sTBS. However, in the rmMANOVA including the other ROIs, we observed no significant impact of the stimulation. This may be due to rather small effects to be detected and potential power problems. Our sample size calculation was based on only one study investigating the effect of TBS on stress-reactive rumination^10^. Please note that we focused on the analysis of the arithmetic task of the TSST. It would further be interesting to analyze the speech phase of the TSST, despite our expectation of similar TBS effects as observed during the arithmetic task. While the continuous nature of the speech task poses methodological challenges—such as the lack of discrete trials for within-subject comparisons—it could provide additional insights into the neurophysiological responses to stress. 

What is particularly interesting is that, contrary to expectations, we did not observe the well-documented prefrontal hypoactivation under stress^7,42^. These findings contrast previous studies with the same experimental setup, but without TBS^43–45,63^. One potential explanation might be the influence of performance-related expectancy effects. As the prefrontal hypoactivation in the TSST has been found to be linked to performance^42 ^it is plausible that participants who perceived TBS as enhancing their performance—regardless of the actual neurobiological effects of TBS—may have experienced improved cognitive output during the TSST. This could have led to the attenuation or absence of the typical prefrontal hypoactivation. We also suggest that expectancy effects might have an impact on the assessed (psychological) variables because the order of stimulation conditions had a significant impact on most variables and several effects were only observed at the first appointment. 

One factor contributing to the observed patterns may be participants’ expectations regarding the impact of TBS on their performance. In exploratory analyses, these beliefs appeared to be associated with stress and negative affect, either through interactions with time or as main effects. However, since expectancy was not systematically manipulated in our study design, only investigated in an exploratory analysis, and given that subgroups became very small, these findings should be interpreted with caution. 

Further, although we recruited primarily naive participants, 65–85% identified their stimulation condition at the second appointment. Regardless of the potential role of expectancy effects, the main finding of the current investigation is a strong habituation to the TSST in the neural stress response and stress-reactive rumination. 

Another possible source of inconsistent findings is the strong hemodynamic response induced by the TSST. The small TBS-effects might have vanished due to the intensity of the stressor. This might also be reflected on a cognitive level as reappraisal as an emotion-regulation strategy is preferred following low-intensity stimuli^64,65^. Likewise, TBS-effects on a behavioral level might be obliterated. Note that we did not observe prefrontal hypoactivation in high ruminators which has repeatedly been found in depression^7^. This contrasts recent findings that aberrant prefrontal functioning has been observed in high ruminators as trait rumination and depressive symptoms are highly correlated and similar neural activation was observed^42^. 

Unfortunately, the fNIRS-device used does not allow coil placement and concurrent measurement and only records relative hemoglobin concentration changes, based on a baseline measurement taken at the start of each segment. This means that brain activity could not be recorded during stimulation. 

Lastly, one likely contributor to the inconsistency of findings is the increasingly questioned assumption that TBS protocols have inherently excitatory or inhibitory effects. This classification, originally derived from studies targeting the motor cortex, may be an oversimplification. This is supported by findings of increased cortical oxygenation following cTBS and decreased oxygenation following iTBS in some channels. A recent meta-analysis by Kirkovski et al.^66^ concluded that both iTBS and cTBS can produce either excitatory or inhibitory effects on neural activity and connectivity, particularly when applied to the prefrontal cortex. In addition to methodological variability (e.g., targeting strategies, coil placement), this heterogeneity likely reflects the complex neural architecture of the frontal cortex and contextual influences, like task demands. Accumulating evidence suggests that individual differences in psychological states may correlate with the magnitude and directionality of effects of NIBS^67^ and individual characteristics (age, genetics) have a substantial impact^68,69^. This emerging understanding challenges the traditional classification of protocols as purely excitatory or inhibitory and highlights the need for more nuanced models^70^. 

Future research should carefully consider within-subject designs, which may introduce habituation and comparability issues across sessions. Systematic investigation of factors influencing variability in TBS effects—such as task demands and cognitive load—is recommended, with an emphasis on standardizing task engagement and avoiding stimulation without concurrent cognitive tasks. Importantly, neural correlates must be measured, as behavioral null results do not imply absent neural effects. Advances in methods enabling neural activity assessment during stimulation should be adopted to enhance mechanistic understanding and interpretability in TBS research.

## Methods

### Participants

Participants were recruited by sending informational emails to mailing lists within the university, reaching over 50,000 individuals. Approximately 1000 expressed interest, and 850 completed an online screening assessing the Ruminative Response Scale (RRS)^71^ as well as demographic and clinical variables. Inclusion and exclusion criteria (see supplementary material S4) were checked again in a telephone interview. To determine the required sample size, we conducted an a priori power analysis using G*Power for a 2 × 2 mixed design (Group × Time), targeting the detection of a between-within interaction effect (see supplementary material S5). Accordingly, we aimed for a sample size of 88 subjects comprising 44 low and 44 high ruminators based on their scores on the online RRS. Specifically, low ruminators were defined as individuals with a mean RRS score of ≤ 1.82, corresponding to the 25th percentile rank, and high ruminators as individuals with a mean RRS score of ≥ 2.36, corresponding to the 64th percentile rank. These cutoffs were determined based on normative data from a large sample previously assessed by our group (*N* = 983)^44,45^. We recruited participants until this target was reached. Please note that we accidentally recruited one additional male high ruminator receiving sTBS at his first and iTBS at his second appointment.

In total, 139 individuals were recruited, however 23 declined participation and 27 participants were excluded as their RRS scores, assessed one week prior to or at their first laboratory appointment, no longer clearly qualified them as either high or low trait ruminators (i.e., if they switched groups or scored closer to the opposite group) (for a CONSORT-diagram, see supplementary material S6). The final sample consisted of 89 right-handed healthy volunteers aged 18 to 50 years (68.54% female, *M*_age _= 23.72 years, *SD*_age _= 6.01 years).

After inclusion, participants were randomly assigned to either intermittent TBS (“iTBS-arm”, *n* = 45; of these, 22 were low ruminators and 23 were high ruminators) or continuous TBS (“cTBS-arm”, *n* = 44; of these, 22 were low ruminators and 22 were high ruminators). Each participant attended two laboratory appointments during which they received either active stimulation (iTBS or cTBS) or sham stimulation (sTBS). The order of sham and active stimulation was randomized and balanced across participants and stimulation groups.

### Procedure

**Fig. 8:**
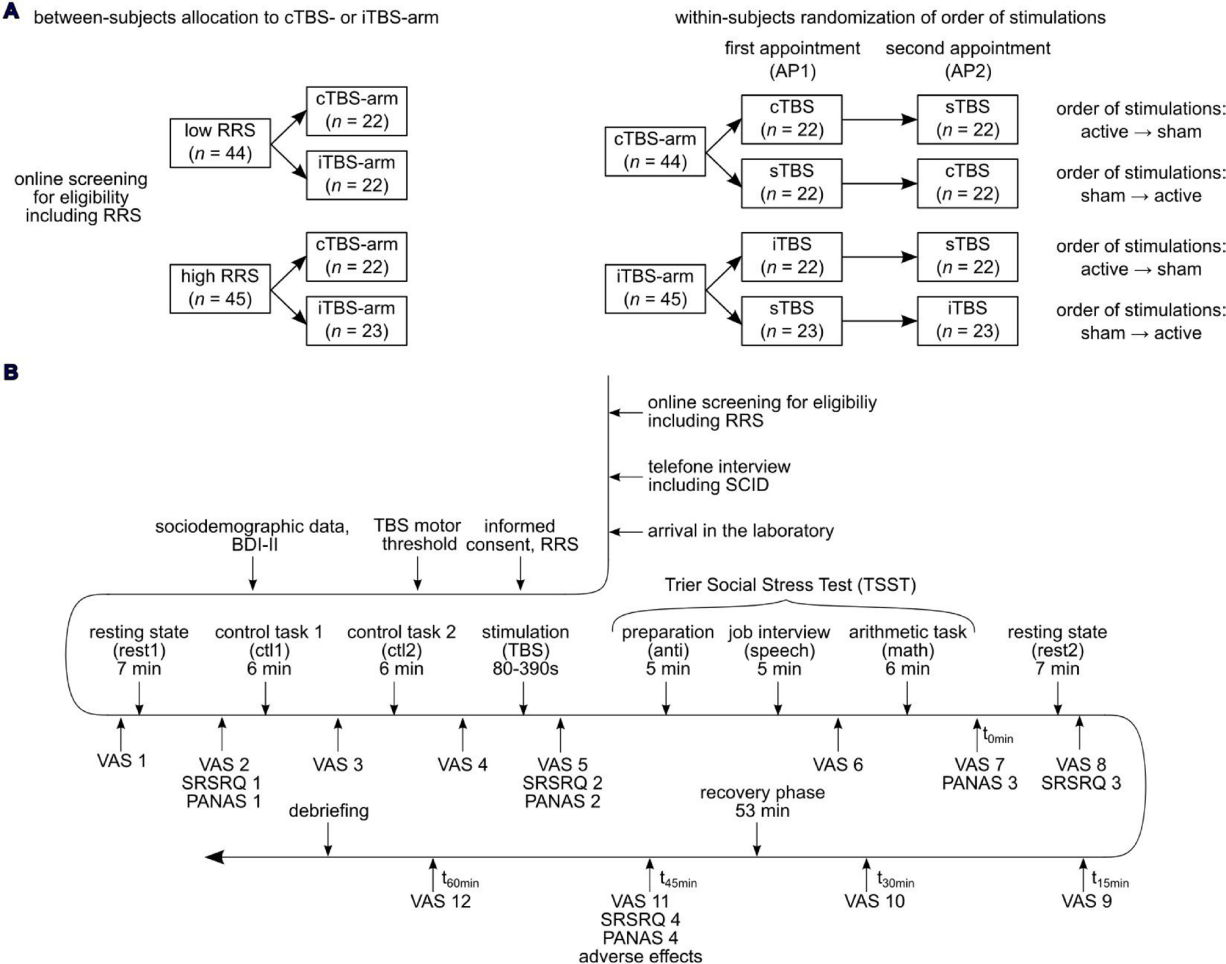
Overview over the study design (**A**) and time course of the experimental procedure (**B**). *RRS =* Ruminative Response Scale, *SCID =* Structured Clinical Interview, *TBS* = Theta Burst Stimulation, *BDI-II =* Beck’s Depression Inventory II, *VAS =* Visual Analogue Scale assessing subjective stress, *SRSRQ =* Stress-Reactive State rumination Questionnaire, *PANAS =* Positive and Negative Affect Schedule.

The two appointments were scheduled approximately 5 weeks apart in order to reduce the memory effects of the experimental procedure and stimulation (*M* = 41.30 days, *SD* = 8.28 days). At the first appointment, subjects gave written informed consent, and completed the RRS again. Afterwards, the participants’ individual resting motor threshold was determined. Then, functional near-infrared spectroscopy (fNIRS) and electrocardiography measurements (ECG) were prepared while the subject had to complete several questionnaires assessing socio-demographic data and depressive symptoms using the Beck Depression Inventory II (BDI-II)^61^. Throughout the entire experiment, subjects rated their subjective stress level at the moment on a Visual Analog Scale (VAS) ranging from 0 to 100% 12 times (Fig. 8). After completing the above-mentioned questionnaires, the first stress rating was assessed (VAS 1; baseline). A 7-min resting-state followed (rest1), where participants were instructed to let their mind wander while keeping their eyes open. Then, another stress rating was assessed (VAS 2; post rest1) as well as current mood using the Positive and Negative Affect Schedule (PANAS)^72^ (PANAS 1; post rest1) (note that an adapted version with 2 additional items was used, namely “happy” and “sad”) and state rumination using the Stress-Reactive State rumination Questionnaire (SRSRQ)^73^ (SRSRQ 1; post rest1) (items in supplementary material S7). Then, two control tasks followed with a similar structure to the arithmetic task of the Trier Social Stress Test (TSST), which were intended as control conditions without social stress and time pressure. For the first control task (ctl1), subjects were given a sheet of paper with number sequences to be read out loud in a total of six trials. During control task 2 (ctl2), also comprising 6 trials, subjects were given different starting numbers from which they should continuously subtract 13 or 17 (depending on the appointment, randomized in order). Each trial consisted of 40s reading and 20s inter-trial interval. In case subjects made an error in ctl2, they had to start over again from the respective starting number, while this was not the case for ctl1. After each task, subjective stress ratings were assessed (VAS 3; post ctl1 and VAS 4; post ctl2) and following both control tasks, the fNIRS- and the ECG-assessments were interrupted, and the neurostimulation was performed in a separate room. The fNIRS-device did not allow for simultaneous measurement of cortical oxygenation during neurostimulation, as the coil placement would have interfered with the optode positioning on the head. After the stimulation, participants gave another stress rating (VAS 5; post stimulation) and returned to the fNIRS room. Five min after the stimulation, they were asked to complete another PANAS (PANAS 2; post stimulation) and SRSRQ (SRSRQ 2; post stimulation). Meanwhile, the fNIRS and ECG were prepared. Readjusting the probeset, which involved removing the participants’ hair from beneath the optodes, took between 5 and 10 min depending on the participant’s hair type. Then, the stress induction using the TSST followed (for details see supplementary material S8), consisting of a 5 min anticipation phase where participants were allowed to take notes, a 5 min job interview where they had to give a speech about their strengths and qualifications, and a 5 min arithmetic task. Following both tasks, participants rated their stress (VAS 6; post speech and VAS 7; post math) and directly after the TSST, another PANAS was assessed (PANAS 3; 0 min post TSST). Then, another 7 min resting-state was conducted (rest2) and subjects completed another SRSRQ (SRSRQ 3; post rest2). Subjective stress was assessed every 15 min for 1 h after the TSST (VAS 8–12), and at 60 min after the TSST subjects additionally completed a fourth SRSRQ (SRSRQ 4; 60 min post TSST) and PANAS (PANAS 4; 60 min post TSST) and adverse effects of the TBS were assessed. Further, we assessed the subjects’ expectancy effects using a custom questionnaire. More specifically, they were asked about the stimulation condition they were in (sham vs. active), whether they believed the stimulation made them perform better vs. worse vs. had no impact on their performance during the TSST and the confidence about their respective answers in percent.

After completing both appointments, participants were debriefed and received either monetary compensation (100 €) or 6 h of course credit. This study was approved by the ethics committee at the University Hospital and University of Tübingen (673/2019BO1). All methods were carried out in accordance with relevant guidelines and regulations.

### Theta Burst Stimulation (TBS)

A study nurse, not further involved in the study, performed the motor threshold determination and stimulation. We used a MagVenture MagPro X100 Stimulator (MagVenture, Farum, Denmark), a figure-eight shaped coil without cooling for motor threshold determination (MagVenture C-B60 coil) and another figure-eight-shaped coil with active cooling (MagVenture Cool-B65 Active/Placebo coil) for the stimulation. This design enables double-blind stimulation by automatically switching between active or sham TBS by coil flipping as per the device instructions. Motor threshold was determined using an electromyogram (EMG) with the 4-channel EMG-EP-system (Schreiber & Tholen Medizintechnik GmbH, Stade) (for details, see supplementary material S9). The TBS over the right VLPFC was applied at 80% of the resting motor threshold^2^. Stimulation was applied at the 10–20 electrode position F8, which corresponded to the maximum of the induced electric field. The induced electric field is relatively focal, as the Magventure B-65 coil used has a half-value depth of 1.4 cm^74 ^meaning the signal intensity decreases by 50% after penetrating 1.4 cm into the tissue. This ensures that the right VLPFC is appropriately targeted (for an illustration of the approximation of the TBS-induced electric field see supplementary material S9). After disinfecting the corresponding skin area, two pre-gelled surface electrodes (28 × 20 mm) were placed 1 cm around the stimulation site to induce a low current for a superficial sensation during stimulation (active and sham). Three different TBS protocols were utilized in the study: iTBS (40 cycles of 2 s theta burst trains (10 bursts of 3 pulses each) followed by 8 s of rest (i.e., total of 390 s), cTBS (80 s train of uninterrupted TBS including 400 bursts of 3 pulses at a frequency of 50 Hz and burst frequency of 5 Hz) and sham stimulation, where the duration was the same as either the iTBS or cTBS protocol. Consequently, the stimulation parameters included a total of 1200 pulses for both paradigms.

### Electrocardiogram (ECG)

After disinfecting the corresponding skin areas, three Ag/AgCl ring electrodes with an 8 mm diameter were placed below the left costal arch, above the right collar bone, and below the neck (reference). The signal was assessed using a BrainAmp ExG amplifier and BrainVision recorder software (Brain Products, Munich, Germany) with a sampling rate of 1000 Hz. Preprocessing using BrainVision Analyzer 2.1 involved band-pass filtering (1–30 Hz; slope: 48 db/Oct, time constant 0.1591549 s) and a notch filter at 50 Hz to eliminate power line artifacts. Using MATLAB 2017a, we then computed the mean interval between consecutive R-peaks in beats per minute (BPM) for each experimental condition.

### Near-infrared spectroscopy (fNIRS)

Cortical oxygenation was assessed using an ETG-4000 Optical Topography System with a sampling rate of 10 Hz (46-channel continuous wave multichannel fNIRS system; Hitachi Medical Co., Japan). Two frontal probesets and one parietal probeset were integrated into EEG-Easycaps, which were placed on the subject’s head oriented to the electrode positions Fpz and Cz. Probeset placement and regions of interest (ROI) are illustrated in supplementary material S10. We stimulated electrode position F8 of the 10–20 system^75 ^which corresponded to one channel of our right frontal probeset. For details on the preprocessing of the fNIRS data using custom MATLAB2024 scripts, we refer to supplementary material S10. For data analysis, event-related averages were computed for each 40 s trial of ctl1, ctl2 and the arithmetic task of the TSST including a 5 s baseline correction. Data were exported separately for the stimulated channel and as an average for each of our ROIs: Left and right VLPFC, left and right DLPFC, and SAC. We chose fNIRS to assess the neural correlates, specifically cortical oxygenation, because it is relatively robust to motion artifacts (see e.g. review by Pinti et al.^76^), which are common during the TSST (e.g., gestures and facial expressions). Unlike fMRI, where participants must remain still, fNIRS allows participants to move naturally, enhancing ecological validity and ensuring higher similarity to the original TSST when fNIRS is employed^77^. Additionally, fNIRS has been successfully used in conjunction with TMS to measure TMS-induced neural changes^78^.

### Data analysis

Data analysis was performed using SPSS (Version 28, IBM Corp., 2021). Data visualization was carried out using MATLAB 2024, RStudio Version 2022.02.3 + 492, R Version 4.3.1 and the ggplot2 package. 

We firstly identified and excluded multivariate outliers using Mahalanobis distances for each of our dependent variables (DV): subjective stress, state rumination, positive and negative affect, math performance, heart rate, and cortical oxygenation (for the number of excluded participants per DV, see supplementary material S11). Then, to analyze the effects of TBS, we fitted repeated measures ANOVAs (rmANOVAs) using contrasts comparing active stimulation (iTBS/cTBS) with sham stimulation before performing planned contrasts (see below). For an analysis of the raw data, we refer to supplementary material S12. Significant effects were followed by post-hoc tests, which were corrected for multiple comparisons using the Benjamini-Hochberg procedure. Non-significant post-hoc tests and those related to lower-order effects in significant higher-order interactions are not reported. Polynomial contrasts (linear and quadratic) were used for interpretative purposes, particularly to capture potential nonlinear changes, such as a peak during stress induction followed by a return to baseline in the post-stress phase. 

Violations of sphericity (Mauchly test *p* < .05) were corrected using Greenhouse-Geisser estimates if $\epsilon$ < 0.75, and Huynh-Feldt estimates if $\epsilon$ > 0.75.

There were no significant baseline differences between the stimulation conditions. 

1. **Contrast analysis**: For each DV, we calculated contrasts by subtracting the sham stimulation values from the active stimulation values at each time point. These contrasts were then entered into rmANOVAs which included a four-way interaction of time, stimulation condition (iTBS vs. cTBS), group (low vs. high RRS), and the order of stimulation conditions (active → sham vs. sham → active). Time refers to the number of repeated assessments for each DV (12 for subjective stress, 4 for state rumination, 4 for positive and negative affect, 3 for math performance, 7 for heart rate, and 3 for cortical oxygenation). To account for potential habituation effects in stress responses due to repeated exposure to the TSST, we included the factor “order of stimulation conditions” in the rmANOVA. This allowed us to better capture how habituation may interact with the effects of stimulation on stress reactivity. When interactions involving the order of conditions were observed, we conducted separate rmANOVAs to analyze each order individually. 
2. **Planned contrasts**: Finally, based on our previous studies^43–45^—using the same experimental design but without neurostimulation—where we investigated on the one hand high and low trait ruminators and on the other hand depressed patients and healthy controls, we conducted planned contrasts to examine specific time points where TBS effects were anticipated to be most pronounced. These studies consistently showed most pronounced group differences for subjective stress and negative affect immediately following the stress induction, while state rumination peaked after the second resting state. Furthermore, we consistently observed prefrontal hypoactivation during the arithmetic task in high-ruminators and depressed patients. Therefore, only these specific time points were selected as planned contrasts. This approach enabled more focused hypothesis testing and increased statistical power. Specifically, we investigated subjective stress ratings following the TSST (post-math, post-rest2, 15–60 min post TSST), state rumination ratings after the second resting state, negative affect ratings following the TSST, and cortical oxygenation in the right VLPFC during the arithmetic task of the TSST.
  We chose the arithmetic task to investigate cortical oxygenation rather than the TSST job interview because, although both are cognitively demanding and we expect similar effects of TBS, the arithmetic task includes non-stressful control conditions (control task 1 and control task 2) with a consistent structure of six trials each that can be averaged. This design allows for within-subject comparisons that are not feasible during the continuous 5-minute job interview. 
  It is important to note that these one-sided *t*-tests are uncorrected for multiple comparisons and should therefore be interpreted with caution and only in conjunction with the broader pattern of findings. 
  To reduce the article’s length, results on positive affect, math performance, and heart rate are included in supplementary material S13–16. 

### fNIRS data

For the fNIRS data, we first conducted a manipulation check using paired *t*-tests to compare cortical oxygenation in each channel during the arithmetic task of the TSST, contrasting active stimulation with sham stimulation (active vs. sham) within subjects across all participants.

Next, we conducted a rmANOVA where cortical oxygenation of the single channel, corresponding to the stimulated electrode position F8, was the DV. Then, a repeated measures MANOVA (rmMANOVA) was performed based on five ROIs (left VLPFC, left DLPFC, right VLPFC, right DLPFC, and SAC), incorporating the aforementioned four-way interaction (see (1) Contrast analysis). 

### Reliable change indices

Additionally, we calculated Reliable Change Indices (RCIs) for state rumination ratings to assess how many participants exhibited statistically meaningful changes between post rest1 and post rest2. We focused the RCI analysis on state rumination, as it represents the primary dependent variable in this study. RCIs are a method used to determine whether an observed change in an individual’s score over time exceeds what could be expected due to measurement error or natural variability alone. In this context, calculating the RCI allows us to identify participants whose changes in state rumination following the stressor are not only statistically significant at the group level but also clinically or psychologically meaningful at the individual level. This approach provides a more nuanced understanding of the effects of the TSST by revealing patterns of reliable individual change that may be masked in group-level analyses. For further details on the application and interpretation of RCI, see ^79^.

### Exploratory analysis

As an exploratory analysis, we also examined the influence of expectancy effects on the previously described raw data analysis. For this purpose, we included the item “Do you believe that the stimulation made you perform better or worse on the task? (better vs. worse vs. no effect)” as a covariate.

## Supplementary Information

Below is the link to the electronic supplementary material.

Supplementary Material 1

## Data Availability

Data is available from the first and last author upon reasonable request.
