# Supplementary material for: Repeated theta burst stimulation of the right ventrolateral prefrontal cortex reveals strong habituation in the context of stress and rumination
Source: Sci Rep. 2025 Aug 23;15:31073. doi: 10.1038/s41598-025-15099-1 (PMC12375055; doi:10.1038/s41598-025-15099-1)
Supplement: Supplementary file 1 — Supplementary Material 1 [file 41598_2025_15099_MOESM1_ESM.pdf]

Supplementary material to

Repeated Theta Burst Stimulation of the Right Ventrolateral Prefrontal Cortex Reveals Strong Habituation in the Context of Stress and Rumination

Isabell Int-Veen[1], Cosima Eisenlohr[2], Ramona Täglich[1], Betti Schopp[1], Hans-Christoph Nuerk[1,2,3,7], Christian Plewnia[1,3], Stefanie De Smet[4,5,6], Marie-Anne Vanderhasselt[4,5], Agnes Kroczek[1], Beatrix Barth[1,3,7], Andreas J. Fallgatter[1,3,7], Ann-Christine Ehlis[1,3,7], David Rosenbaum[1]

[1] Tübingen Center for Mental Health (TüCMH), Department of Psychiatry and Psychotherapy, University Hospital Tübingen, Tübingen, Germany
[2] Department of Psychology, University of Tübingen, Germany
[3] German Center for Mental Health (DZPG), partner site Tübingen, Germany
[4] Department of Head and Skin, Psychiatry and Medical Psychology, Ghent University Hospital, Ghent University, Ghent, Belgium
[5] Ghent Experimental Psychiatry (GHEP) lab, Ghent, Belgium
[6] Brain Stimulation and Cognition (BSC) Lab, Department of Cognitive Neuroscience, Faculty of Psychology & Neuroscience, Maastricht University, Maastricht - The Netherlands
[7] LEAD Graduate School & Research Network, University of Tübingen, Tübingen, Germany

Corresponding Author:
Isabell Int-Veen
Calwerstraße 14
72076 Tübingen
Germany
email: isabell.int-veen@med.uni-tuebingen.de

Supplementary material S1: Details on blinding

According to a binomial test, participants were unable to identify sham from active stimulation during the first appointment (see table S1.1). Additionally, there were no significant differences in stimulation intensity between the cTBS (*M* = 43.89, *SD* = 6.93), iTBS (*M* = 40.80, *SD* = 5.20) and sTBS condition (*M* = 42.18, *SD* = 6.06), *F*(2, 175) = 2.872, *p* = .059, η_p^2 = .03.

	percent	95% CI	*p*-value
correct identification of sTBS as sham (AP1)	46.67%	[31.66; 62.13]	0.766
correct identification of iTBS as active (AP1)	36.36%	[17.20; 59.34]	0.286
correct identification of cTBS as active (AP1)	50.00%	[28.22; 71.78]	0.999
correct identification of sTBS as sham (AP2)	65.91%	[50.08; 79.51]	< .05
correct identification of iTBS as active (AP2)	86.36%	[65.09; 97.09]	< .001
correct identification of cTBS as active (AP2)	78.26%	[56.30; 92.54]	< .05
correct identification of sTBS as sham (overall)	56.18%	[45.25; 66.68]	0.289
correct identification of iTBS as active (overall)	61.36%	[45.50; 75.64]	0.174
correct identification of cTBS as active (overall)	64.44%	[48.78; 78.13]	0.073

Table S1.1. Summary of exact binomial tests assessing the blinding of participants (H_0: *p* = .5). sTBS = sham Theta Burst Stimulation, cTBS = continuous Theta Burst Stimulation, iTBS = intermittent Theta Burst Stimulation, AP1 = first appointment, AP2 = second appointment.

We further tested whether participants believed the stimulation had an impact on their performance (better or worse) using a logistic regression model (see table S1.2). Results indicated no significant effect of condition, χ^2(2) = 1.715, *p* = .424.

As a second step, we fitted a linear mixed model dependent on the condition for ratings of "How confident are you in answering the question?" referring to the question about the impact of the stimulation on their performance: We fitted a model with the response to the respective item as DV, stimulation condition as a predictor and separate intercepts per subject. Again, we did not observe a significant main effect of the condition, χ^2(2) = 1.532, *p* = .465.

When fitting the logistic regression model and linear mixed model dependent on the RRS-group, we also observed no significant effect of the group, χ^2(1) = 0.101, *p* = .751 and χ^2(1) = 0.00, *p* = .996, respectively. In the case of the low RRS-group most participants believed that the stimulation had no impact on their performance (41.3% no change, 35.0% better, 23.8% worse), while in the high RRS-group, most participants believed that the stimulation made them better (38.8% no change, 47.1% better, 14.1% worse).

			condition			
			sTBS	cTBS	iTBS	total
impact on performance	no change	count	38	15	13	66
		% within impact on performance	57.6%	22.7%	19.7%	100%
		% within condition	44.7%	36.6%	33.3%	40.0%
		% of all data points	23.0%	9.1%	7.9%	40.0%
	better	count	33	17	18	68
		% within impact on performance	48.5%	25.0%	26.5%	100%
		% within condition	38.8%	41.5%	46.2%	41.2%
		% of all data points	20.0%	10.3%	10.9%	41.2%
	worse	count	14	9	8	31
		% within impact on performance	45.2%	29.0%	25.8%	100%
		% within condition	16.5%	22.0%	20.5%	18.8%
		% of all data points	8.5%	5.5%	4.8%	18.8%

Table S1.2. Absolute and relative frequencies of participants rating the impact of the stimulation on their performance dependent on stimulation condition. sTBS = sham Theta Burst Stimulation, cTBS = continuous Theta Burst Stimulation, iTBS = intermittent Theta Burst Stimulation. Please note that 13 data points were missing as the item was not answered or was answered ambiguously.

Supplementary material S2: Illustration of Reliable Change Indices

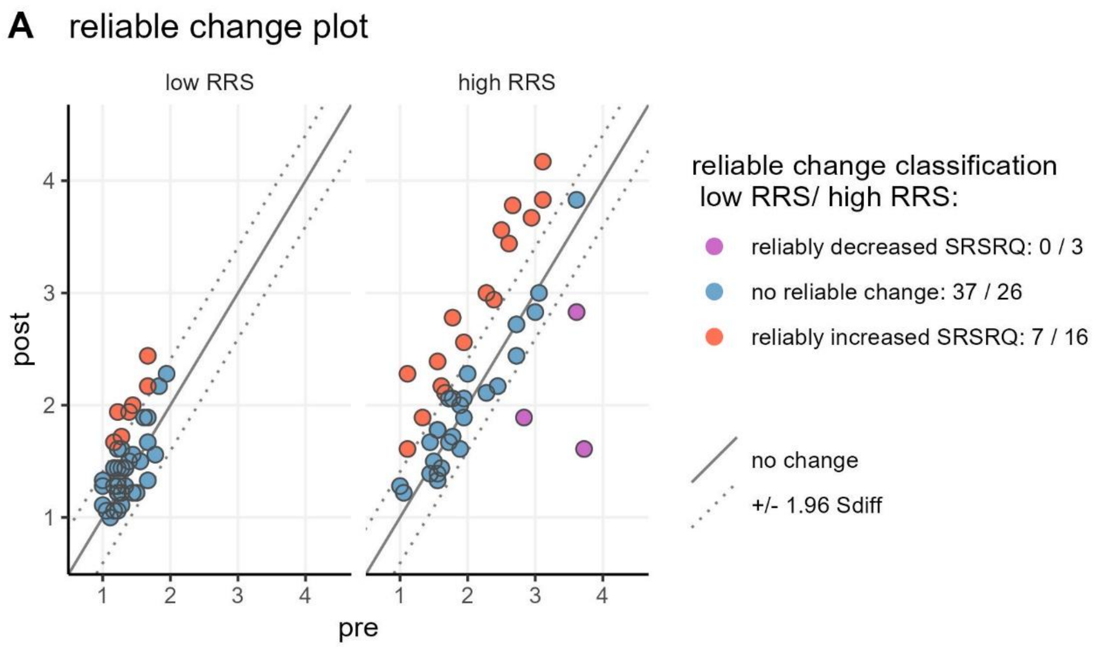

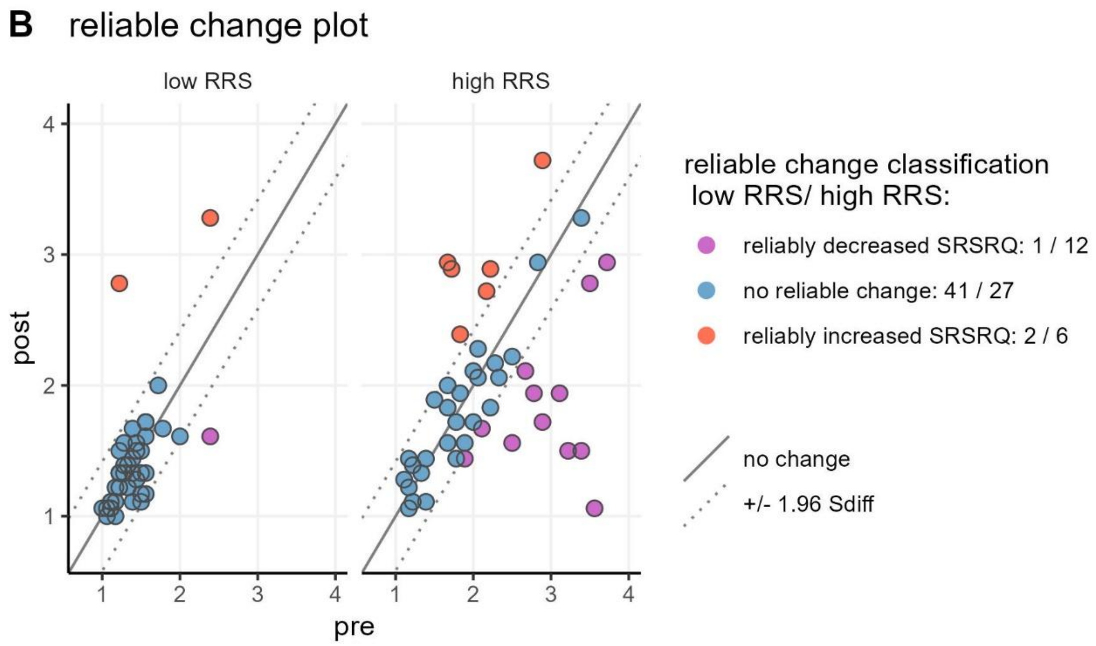

Figure S2. Reliable change indices plotted for changes in state rumination (SRSRQ scores) from before the stress induction (rest1) to after the stress induction (rest2) for the first appointment (A) and second appointment (B) dependent on RRS-group.

Supplementary material S3: Brainmaps

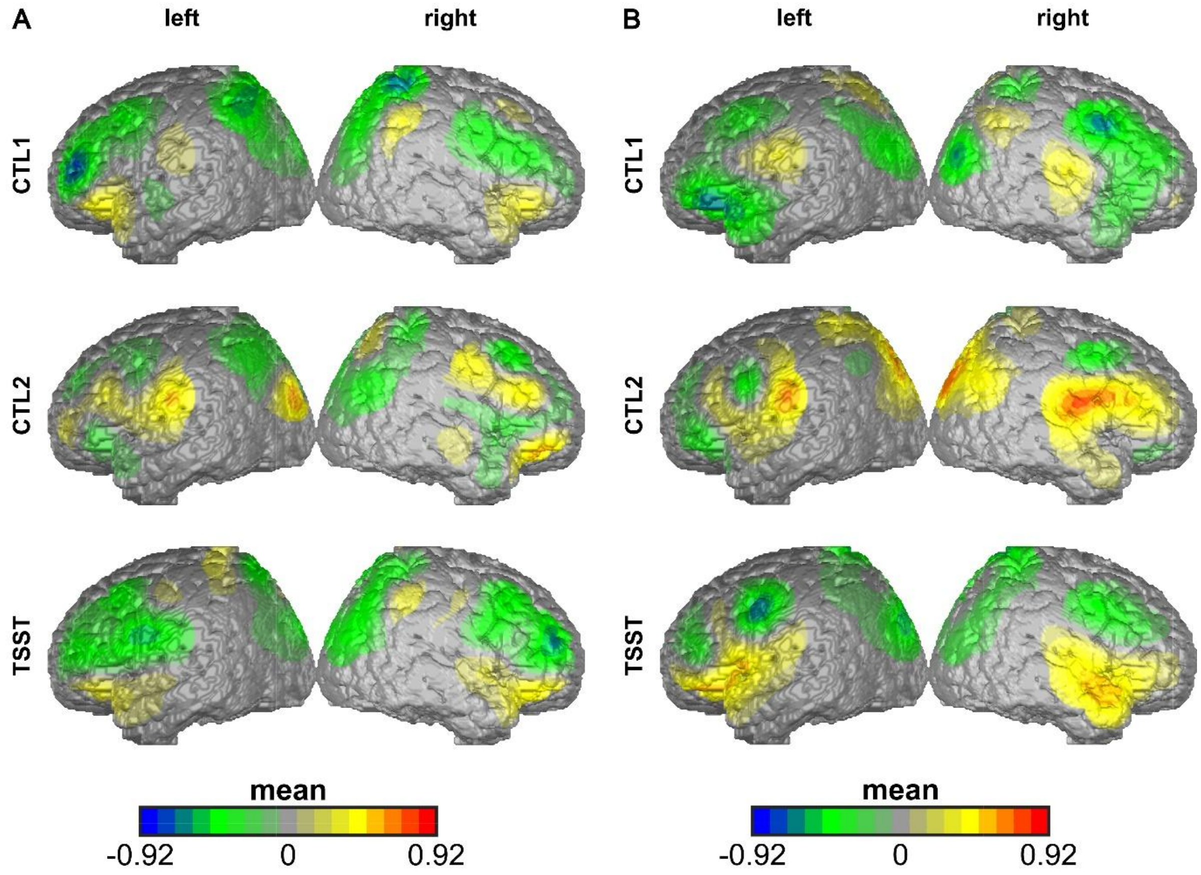

Figure S3.1 Standardized mean cortical oxygenation contrasts (active minus sham stimulation) of low ruminators having received A: cTBS or B: iTBS with the order of stimulation conditions active → sham; CTL1 = control task 1 (reading numbers), CTL2 = control task 2 (performing calculations without social stress); TSST = performing calculations under social stress. Warm colors indicate higher cortical oxygenation following the active stimulation compared to sham stimulation, cool colors indicate higher cortical oxygenation following sham stimulation compared to active stimulation.

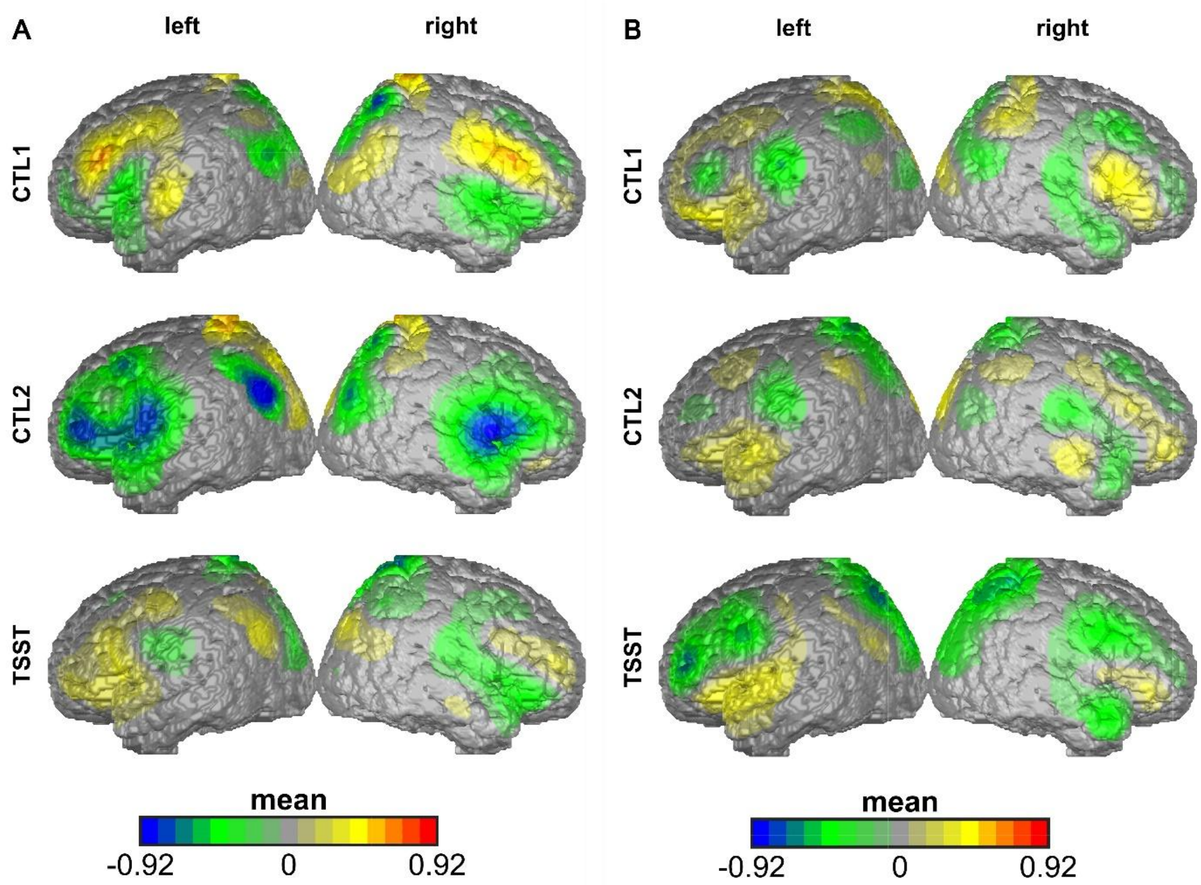

Figure S3.2 Standardized mean cortical oxygenation contrasts (active minus sham stimulation) of low ruminators having received A: cTBS or B: iTBS with the order of stimulation conditions sham → active; CTL1 = control task 1 (reading numbers), CTL2 = control task 2 (performing calculations without social stress); TSST = performing calculations under social stress. Warm colors indicate higher cortical oxygenation following the active stimulation compared to sham stimulation, cool colors indicate higher cortical oxygenation following sham stimulation compared to active stimulation.

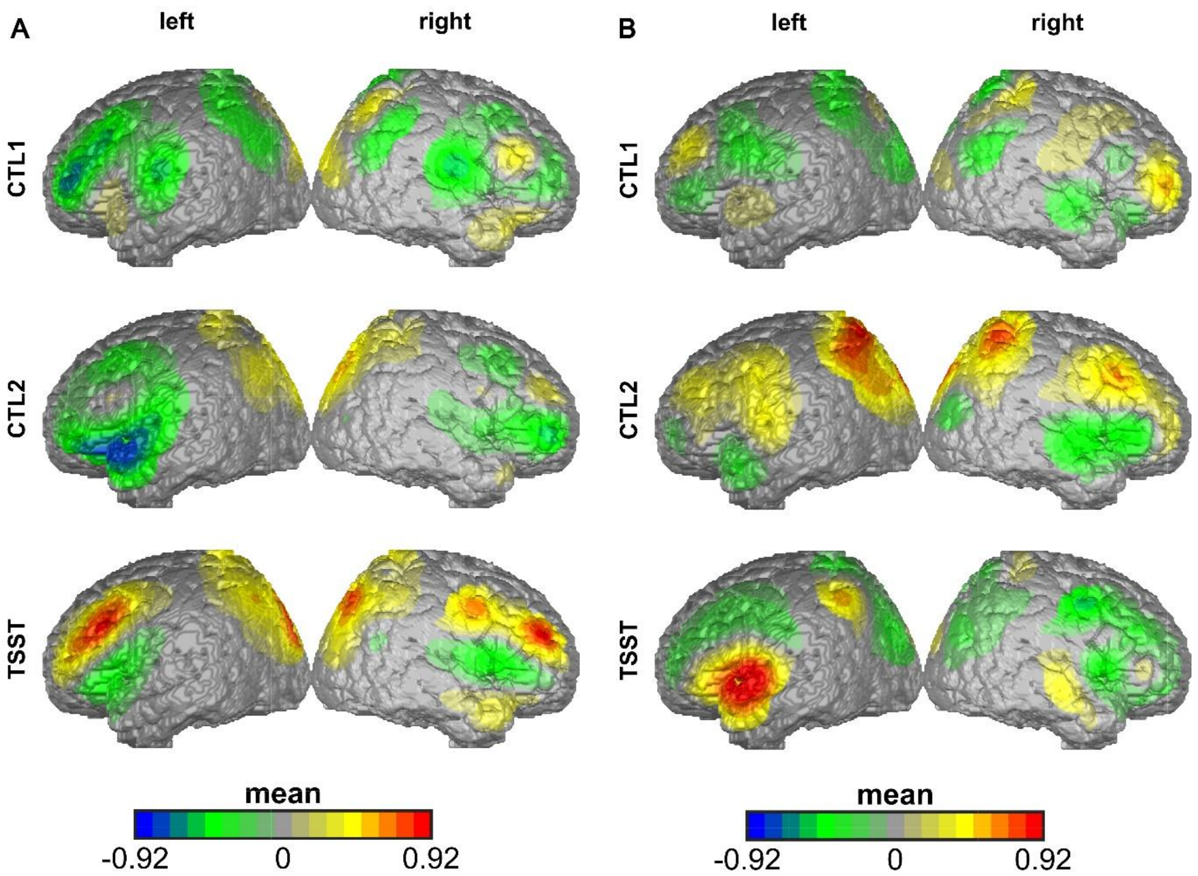

Figure S3.3 Standardized mean cortical oxygenation contrasts (active minus sham stimulation) of high ruminators having received A: cTBS or B: iTBS with the order of stimulation conditions active → sham; CTL1 = control task 1 (reading numbers), CTL2 = control task 2 (performing calculations without social stress); TSST = performing calculations under social stress. Warm colors indicate higher cortical oxygenation following the active stimulation compared to sham stimulation, cool colors indicate higher cortical oxygenation following sham stimulation compared to active stimulation.

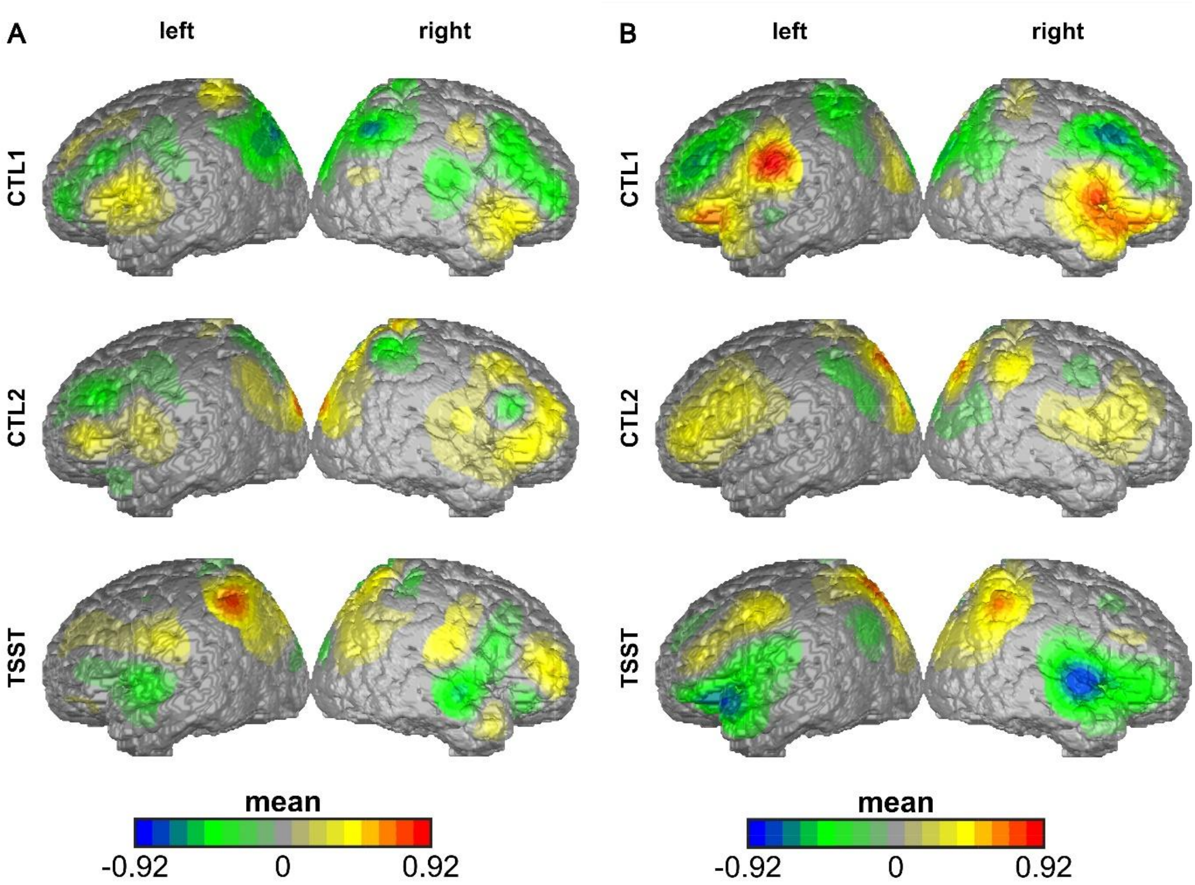

Figure S3.4 Standardized mean cortical oxygenation contrasts (active minus sham stimulation) of high ruminators having received A: cTBS or B: iTBS with the order of stimulation conditions sham → active; CTL1 = control task 1 (reading numbers), CTL2 = control task 2 (performing calculations without social stress); TSST = performing calculations under social stress. Warm colors indicate higher cortical oxygenation following the active stimulation compared to sham stimulation, cool colors indicate higher cortical oxygenation following sham stimulation compared to active stimulation.

Supplementary material S4: Inclusion and exclusion criteria

Inclusion criteria:

- Age between 18 and 50 years
- Normal vision (or appropriate correction)
- Right-handedness
- No metal in the skull / brain
- German as native language or very good knowledge of German

Exclusion criteria:

- Diabetes mellitus
- renal insufficiency
- untreated hypertension
- history of traumatic brain injury
- cardiac arrhythmia
- acute substance abuse
- adrenal insufficiency
- any acute psychiatric or neurological disorder (including any anomalies in the Structured Clinical Interview (SCID)-Screening[1]
- in case of women: pregnancy

Supplementary material S5: Information on the a priori power analysis

Currently, only one study exists that investigated the effects of TBS on state rumination increases through the TSST. In the study of De Witte et al.[2], *N* = 40 participants were either stimulated with iTBS or sham TBS after the TSST was applied. Note that the study also used a within-subject design in which all participants received iTBS and sham after the TSST was conducted on two separate days. In their study, the authors found an increase of state rumination through the TSST and a marginally significant interaction between trait rumination and the TBS protocol on increases of state rumination [2]. The statistical parameters are given as *B* = -.32, *t*(36) = - 1.891, *p* = .067. Thankfully, the authors sent us the data of this study and we therefore were able to compute the effect size of this effect directly from the data, which was η_p^2 = .107 (approx. *d* = .69, *f* = .34). However, it is important to note that the study didn't use a stratified sample of trait rumination or clinical participants. As a result, only 8 of 38 subjects fulfilled our previous criteria for high ruminators[3,4]. As we showed in our previous studies[3–6] that state rumination increased in high trait ruminators/patients more than in low trait ruminators/healthy controls, we would assume that in stratified and clinical samples the above-noted buffering effect of the TBS protocol could be even higher, as the general reactivity of these samples in state rumination (under sham stimulation) is stronger than in the study of De Witte et al.[2].

However, as seen in the study of De Witte et al.[2], it is important to achieve high power as the effect was only marginally significant. Therefore, we aim to assess *N* = 80 subjects which allows to detect between-within subject interactions up to *f* = .15 for the effect of state rumination, as computed with G*Power 3.1.9.2 (with a power of *1-β* = .95, *α* = .05. *r* = .7; between measurements, assessed on the basis of data from our previous studies). To compensate for data loss and drop-outs, we plan to collect an additional 10% (*n* = 8) of participants to the computed sample size, resulting in *N* = 88 participants.

Supplementary material S6: Consort diagram

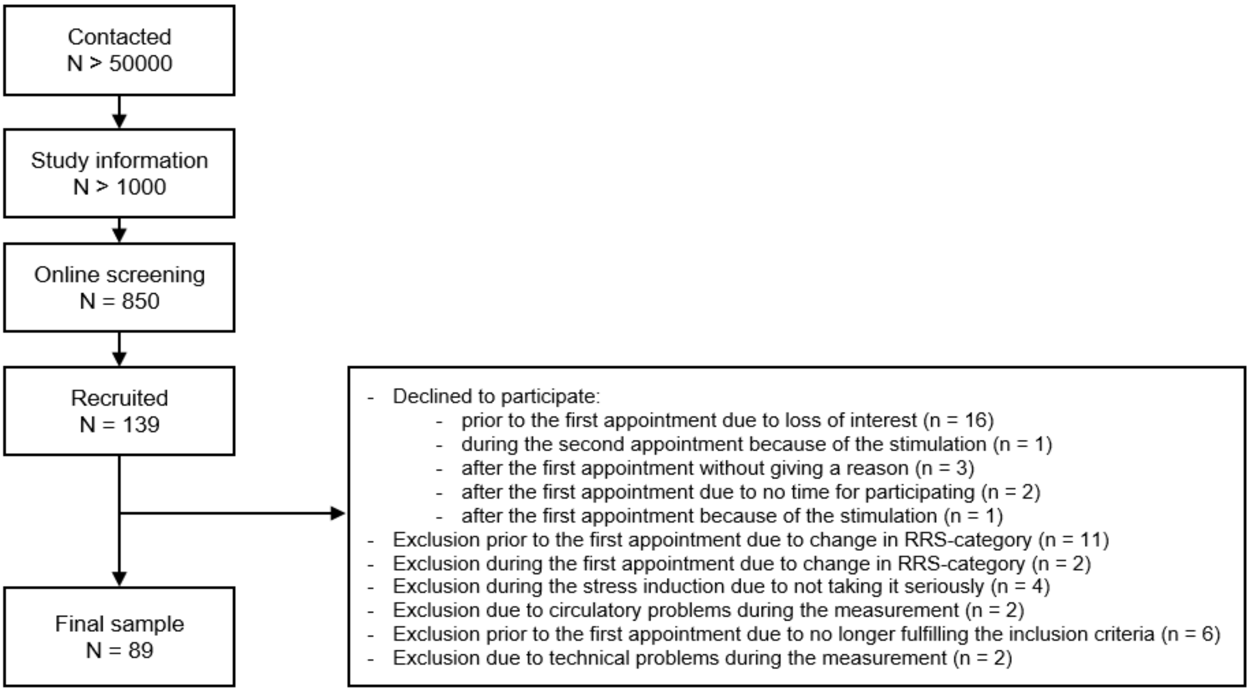

Figure S6. Change in RRS-category = Prior to study inclusion, we screened participants online using the Ruminative Response Scale (RRS)[7] and only recruited low and high ruminators (low trait ruminators: mean RRS ≤ 1.82 (PR 25); high trait ruminators: mean RRS ≥ 2.36 (PR 64)). One week prior to their appointment at the laboratory, participants completed another online RRS. In case they were now categorized as the corresponding other group (low ruminators now exceeding the cutoff of high ruminators or high ruminators now scoring below the low ruminator cutoff) or their score now was closer to the corresponding other group (low ruminators now scoring a medium RRS but closer to the high ruminator cutoff or high ruminators now scoring a medium RRS but closer to the low ruminator cutoff), participants were excluded. At their first appointment in the laboratory, participants completed the RRS a third time, this time in paper-pencil format. Again, the same exclusion criteria as for the second online RRS were applied.

Supplementary material S7: Items of the state rumination questionnaire

Items of the state rumination questionnaire including adapted items from the Ruminative Response Scale[8], Amsterdam Resting-State Questionnaire[9] and the Perseverative Thinking Questionnaire[10]. Subjects were instructed to rate if the items were in line with their mental state during the last resting-state measurement.

item	German	English
1	Ich dachte immer wieder an meine Probleme.	I repeatedly thought about my problems.
2	Ich verharrte im Denken an Dinge, die mich beunruhigen.	I kept thinking about things that bother me.
3	Meine Gedanken wiederholten sich, ohne dass ich zu einer Lösung kam.	I dwelled on my thoughts without coming to a solution.
4	Ich verlor mich in meinen negativen Gedanken.	I got lost in my negative thoughts.
5	Ich konnte meine Gedanken nur mühsam festhalten.	I had difficulties holding on to my thoughts.
6	Ich konnte mich nicht von meinen negativen Gedanken lösen.	I could not let go of my negative thoughts.
7	Ich war bei der Sache.	I was present.
8	Ich dachte darüber nach, warum ich mich in bestimmten Situationen falsch verhalten habe.	I thought about why I acted wrong in certain situations.
9	Ich fragte mich, warum ich Probleme habe, die andere nicht haben.	I thought why I have problems other people don´t have.
10	Ich fragte mich, womit ich meine momentane Lebenssituation verdient habe.	I thought about whereby I deserved my current life situation.
11	Ich dachte darüber nach, warum ich die Dinge nicht besser in den Griff bekomme.	I thought why I can´t handle things better.
12	Ich dachte an all meine Defizite und Misserfolge, Macken und Fehler.	I thought about all my shortcomings, failings, faults, mistakes.
13	Ich konnte flexibel zwischen meinen Gedanken hin und her schalten.	I could switch between my thoughts flexibly.
14	Ich dachte an vergangene Situationen, die ich bereue.	I thought about past situations that I regret.
15	Ich machte mir Selbstvorwürfe.	I blamed myself.
16	Ich verlor mich in Gedanken an Vergangenes.	I got lost in thoughts about the past.
17	Ich war von meinen Problemen und Sorgen stark vereinnahmt.	I was consumed by my problems and worries.
18	Meine negativen Gedanken ließen mich nicht los.	I couldn´t let go of my negative thoughts.

Supplementary material S8: Additional information to the conducted Trier Social Stress Test (TSST)

To induce psychosocial stress, an adaptation of the Trier Social Stress Test (TSST)[11] was used. Participants were informed that two additional people would join for the upcoming tasks before two experimenters wearing white physician coats entered the room and sat down in front of the participant. Then participants were instructed to imagine having applied for a job at the university hospital and having 5 minutes time to prepare a speech about their strengths and qualifications for the respective position. During this anticipation phase (anti) participants were allowed to take notes. After 5 minutes, one of the experimenters started a video camera while the participant was helped to stand up to deliver the speech. The experimenters took away the notes and following this remained socially unresponsive to any social interaction cues. If participants paused for more than 20 s, the experimenters instructed them to continue. After the speech, participants gave another subjective stress rating. Then the experimenter introduced the following arithmetic task which has the same structure as control task 2. Participants had to subtract in either 13 or 17 steps (randomized in order between the two different appointments) from different starting points while holding eye-contact with one experimenter. If participants made a mistake, they had to restart with the initial starting number. The participant is instructed to calculate as fast and correctly as possible while holding eye-contact with one of the experimenters. After the arithmetic task of the TSST the experimenters turn off the camera and leave the room without saying a word, and another stress rating is assessed.

Supplementary material S9: Additional information to section 2.3 Theta Burst Stimulation (TBS)

Previous to the motor threshold determination using a 4-channel EMG-EP-system (Schreiber & Tholen Medizintechnik GmbH, Stade), the corresponding skin areas were sanitized. Then two gold-plated surface cup electrodes of 11 mm diameter were placed on the right abductor pollicis brevis muscle (one thenar and one on the proximal phalanx) and one reference electrode on the inner arm below the wrist. Stimulation intensity was reduced until the amplitude of motor-evoked potentials of 50 µV in less than 50% of 10 consecutive stimuli was recorded.

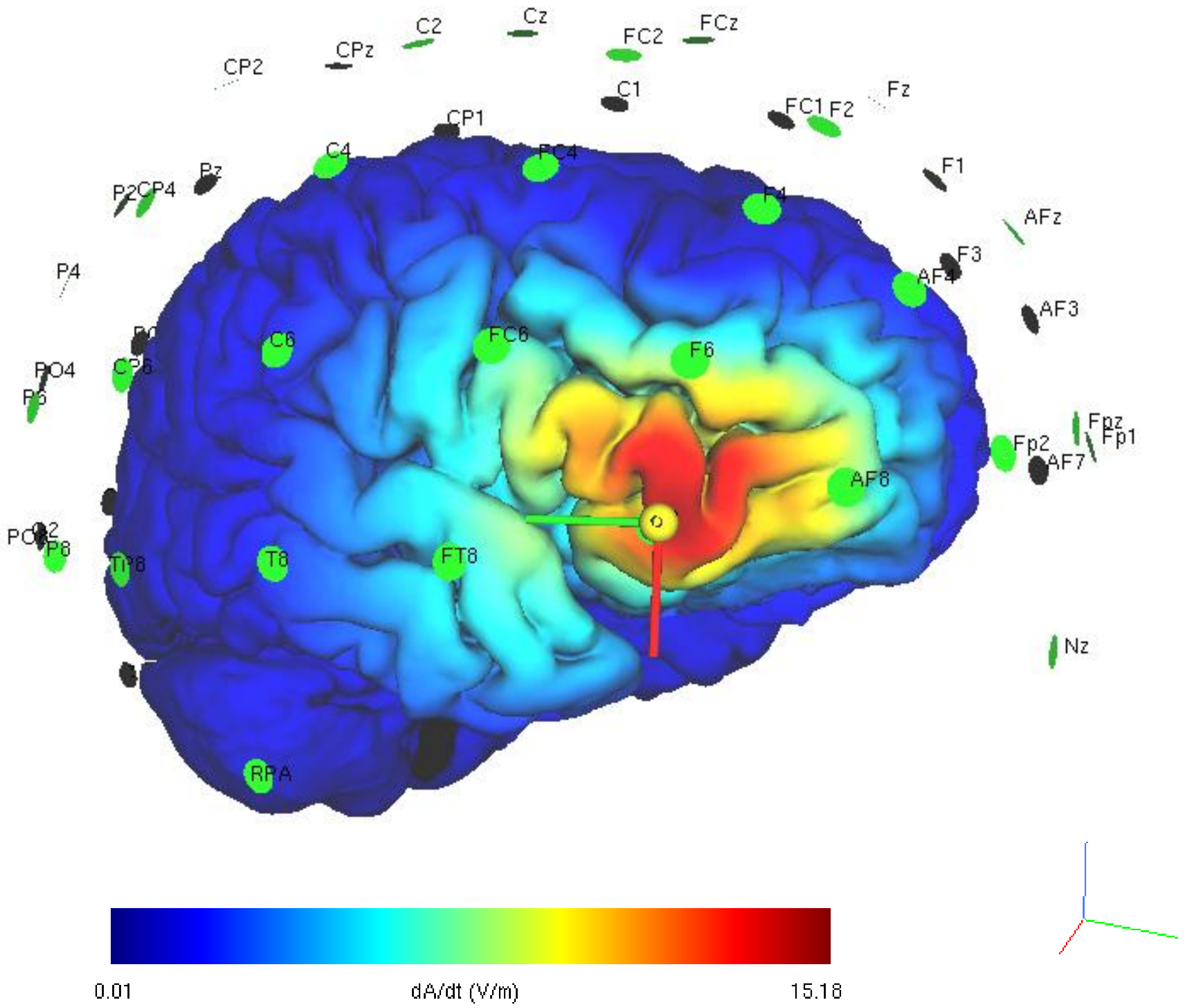

Figure S9. Approximation of the TBS-induced electric field. The coil (MagVenture Cool-B65 Active/Placebo coil) was positioned over the right VLPFC, corresponding to the F8 electrode according to the 10–20 EEG system. The color red indicates the strongest electric field. The y-axis in green indicates the direction of the coil handle. This plot was created using SimNIBS 4.1.0.[12].

Supplementary material S10: Additional information to section 2.5 Near-Infrared Spectroscopy (fNIRS)

In total, we used 28 light emitters (semiconductor lasers) and 15 light detectors (avalanche photodiodes) with an inter-optode distance of 3 cm, operating at two wavelengths (695 ± 20 nm and 830 ± 20 nm). The power output for each wavelength at every optode was 2.0 ± 0.4 mW. We used customized MATLAB 2024 scripts to calculate relative changes in oxygenated (O_2Hb) and deoxygenated (HHb) hemoglobin using the modified Beer-Lambert Law[13]. Preprocessing steps involved an automatic detection and interpolating of single noisy channels, correcting motion artifacts using Temporal Derivative Distribution Repair[14], employing Correlation-based signal improvement[15], and bandpass-filtering to eliminate low-frequency baseline-drifts (< 0.01 Hz) and high-frequency noise (> 0.1 Hz). Then, another channel interpolation followed, and we performed a global signal reduction using a spatial Gaussian kernel filter (σ = 40) and a z-standardization. Note that in the following, O_2Hb data refers to the correlation-based improved O_2Hb signal.

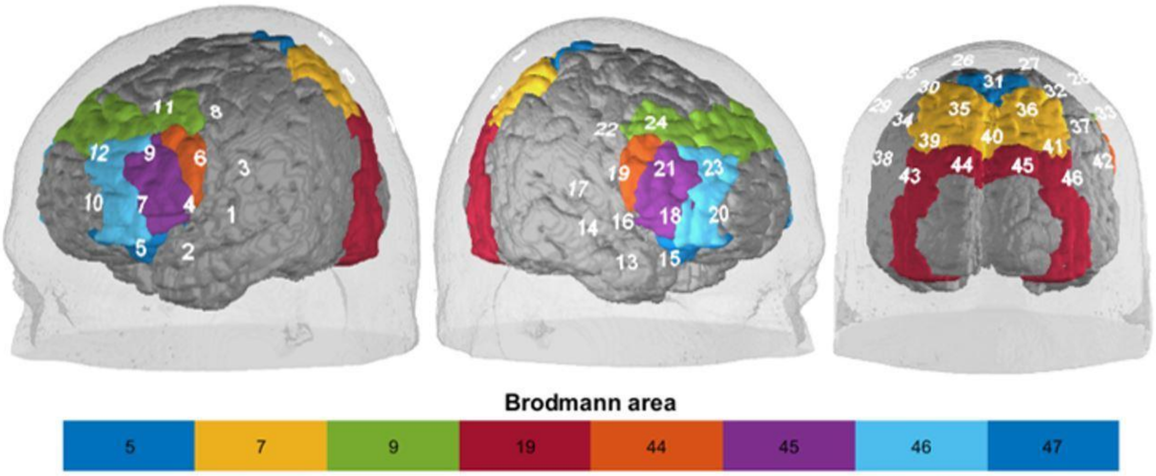

Figure S10. Placement of the three probesets. Scalp-brain correspondence was estimated based on Okamoto et al.[16], Okamoto and Dan[17], and Singh et al.[18]. Please note that the corresponding numbers represent the channels (located midway between emitter and detector).

Table S10: Definition of Regions of Interest

ROI	channel
left VLPFC	7 9 6 5
right VLPFC	18 21 19 15
left DLPFC	10 12 11
right DLPFC	20 23 24
SAC	27 26 25 28 30 31 32 35 36

Note. VLPFC = Ventrolateral Prefrontal Cortex, DLPFC = Dorsolateral Prefrontal Cortex, SAC = Somatosensory Association Cortex. Please note that the TBS was applied over channel 15 which corresponds to F8.

Supplementary material S11: Details on the number of excluded participants

- **Subjective stress contrasts:** Checking for multivariate outliers using Mahalanobis distances ($p < .001$), we excluded the data of 4 participants for the following analysis.
- **Subjective stress raw data:** Checking for multivariate outliers using Mahalanobis distances ($p < .001$), we excluded 12 data points, namely 5 participants' data of the first appointment (AP1) and 7 participants' data of the second appointment (AP2) for the following analysis.
- **State Rumination contrasts:** According to their Mahalanobis distances ($p < .001$), we excluded the data of 2 participants and 2 participants due to missing data from the following analysis.
- **State Rumination raw data:** After checking for multivariate outliers using Mahalanobis distances ($p < .001$), we excluded 7 data points and 2 due to missing data, specifically 5 participants' data from the first appointment and 4 participants' data from the second appointment, for the following analysis.
- **Negative affect contrasts:** Based on their Mahalanobis distances ($p < .001$), the data of 2 participants, and 2 participants due to missing data, were excluded prior to fitting the rmANOVA.
- **Negative affect raw data:** After checking for multivariate outliers using Mahalanobis distances ($p < .001$), we excluded 3 data points and 2 due to missing data, specifically 3 participants' data from the first appointment and 2 participants' data from the second appointment, for the following analysis.
- **Cortical oxygenation contrasts single channel:** No data was flagged as a multivariate outlier ($p < .001$), however, 2 participants had to be excluded due to missing data.
- **Cortical oxygenation contrasts ROIs:** No data was flagged as a multivariate outlier ($p < .001$), however 2 participants had to be excluded due to missing data.
- **Cortical oxygenation raw data ROIs:** No data was flagged as a multivariate outlier using Mahalanobis distances ($p < .001$), however, we excluded 2 missing data, namely one participant's data of the second appointment and one participant's data of the first appointment for the following analysis.

Supplementary material S12: Results of the analysis of the raw data

In addition to the contrast analysis, we analyze raw data to offer a clearer overview of the observed effects. Specifically, we fitted rmANOVAs with a three-way interaction of time, stimulation condition (iTBS vs. cTBS vs. sTBS), and group (low RRS vs. high RRS) for each appointment separately.

Subjective stress raw data

We investigated the raw data using a rmANOVA for subjective stress dependent on the three-way interaction of time (indicating the 12 repeated assessments of subjective stress ratings over the course of one appointment), RRS-group (low vs. high ruminators) and stimulation condition (cTBS vs. iTBS vs. sTBS) separately for each appointment. Here, we again only observed significant main effects of time (AP1: $F(3.407, 265.762) = 95.194$, $p < .001$, $\eta_p^2 = .550$; AP2: $F(2.807, 213.362) = 65.757$, $p < .001$, $\eta_p^2 = .464$). Polynomial contrasts revealed a quadratic time course for both appointments, indicating successful stress inductions during both the first and second exposures (AP1: $F(1, 78) = 194.839$, $p < .001$, $\eta_p^2 = .714$; AP2: $F(1, 76) = 102.062$, $p < .001$, $\eta_p^2 = .573$). At the first appointment, we also observed a significant main effect of RRS-group, $F(1, 78) = 6.076$, $p < .05$, $\eta_p^2 = .072$, indicating higher stress in high ruminators, as well as a significant main effect of stimulation condition, $F(2, 78) = 3.320$, $p < .05$, $\eta_p^2 = .078$, reflecting overall higher subjective stress in case participants received iTBS.

State rumination raw data

Next, we investigated the raw data using a rmANOVA for state rumination dependent on the three-way interaction of time (indicating the 4 repeated assessments of state rumination ratings over the course of one appointment), RRS-group (low vs. high ruminators) and stimulation condition (cTBS vs. iTBS vs. sTBS) separately for each appointment. We observed a significant interaction of time and RRS-group but only at the first appointment: $F(3, 234) = 6.271$, $p < .001$, $\eta_p^2 = .074$. Using polynomial contrasts to investigate this interaction, we found a quadratic association, $F(1, 78) = 4.008$, $p < .05$, $\eta_p^2 = .026$, indicating higher increases in state rumination in the case of the high ruminators compared to low ruminators.

At the second appointment, we did not observe a significant interaction but significant main effects of time, $F(2.885, 227.935) = 6.826$, $p < .001$, $\eta_p^2 = .080$, and RRS-group, AP2: $F(1, 79) = 31.092$, $p < .001$, $\eta_p^2 = .282$, indicating overall higher state rumination in high ruminators compared to low ruminators. Lastly, only when considering polynomial contrasts, we observed a significant quadratic contrast for the interaction of time, RRS-group and stimulation condition at the first appointment, $F(2, 78) = 3.272$, $p < .05$, $\eta_p^2 = .077$. Interpreting this contrast using plots of the raw data, low ruminators exhibited generally low state rumination ratings and only

minimal increases throughout the experimental procedure and regardless of the stimulation condition. Descriptively, high ruminators exhibited steeper decreases in state rumination ratings directly after the stimulation following cTBS but comparable ratings for all other time points. Please note, however, that there were no significant differences between the stimulation conditions before as well as after correction for multiple comparisons.

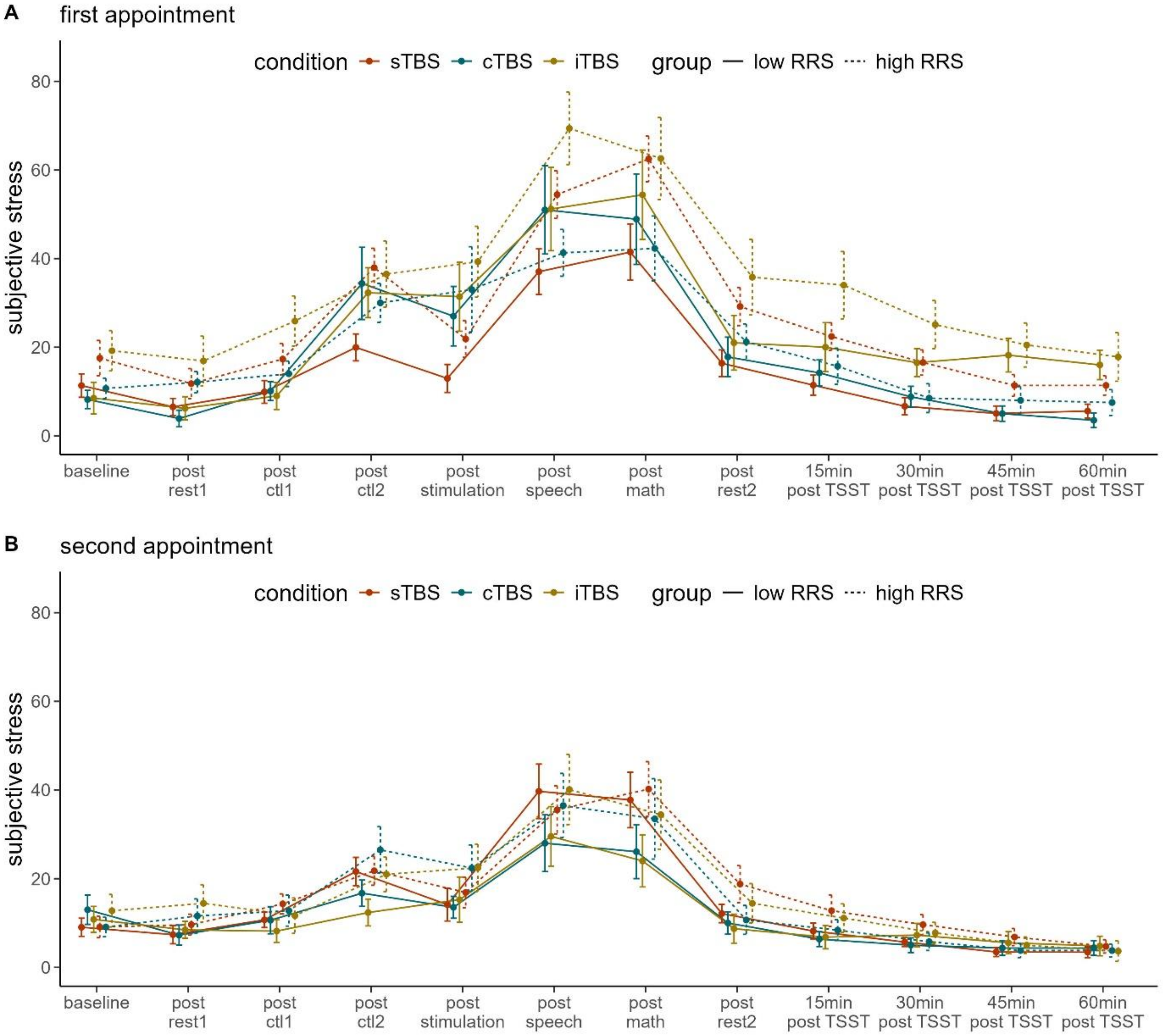

Figure S12.1 Line plot of the raw data of subjective stress ratings dependent on the appointment (A = first appointment; B = second appointment). rest = resting-state measurement, ctl1 = control task 1, ctl2 = control task 2, speech = job interview of the TSST, math = arithmetic task of the TSST, TSST = Trier Social Stress Test, sTBS = sham theta burst stimulation, cTBS = continuous theta burst stimulation, iTBS = intermittent theta burst stimulation, 15 min = 15 min after the TSST, RRS = Ruminative Response Scale. Error bars indicate 1 standard error of the mean.

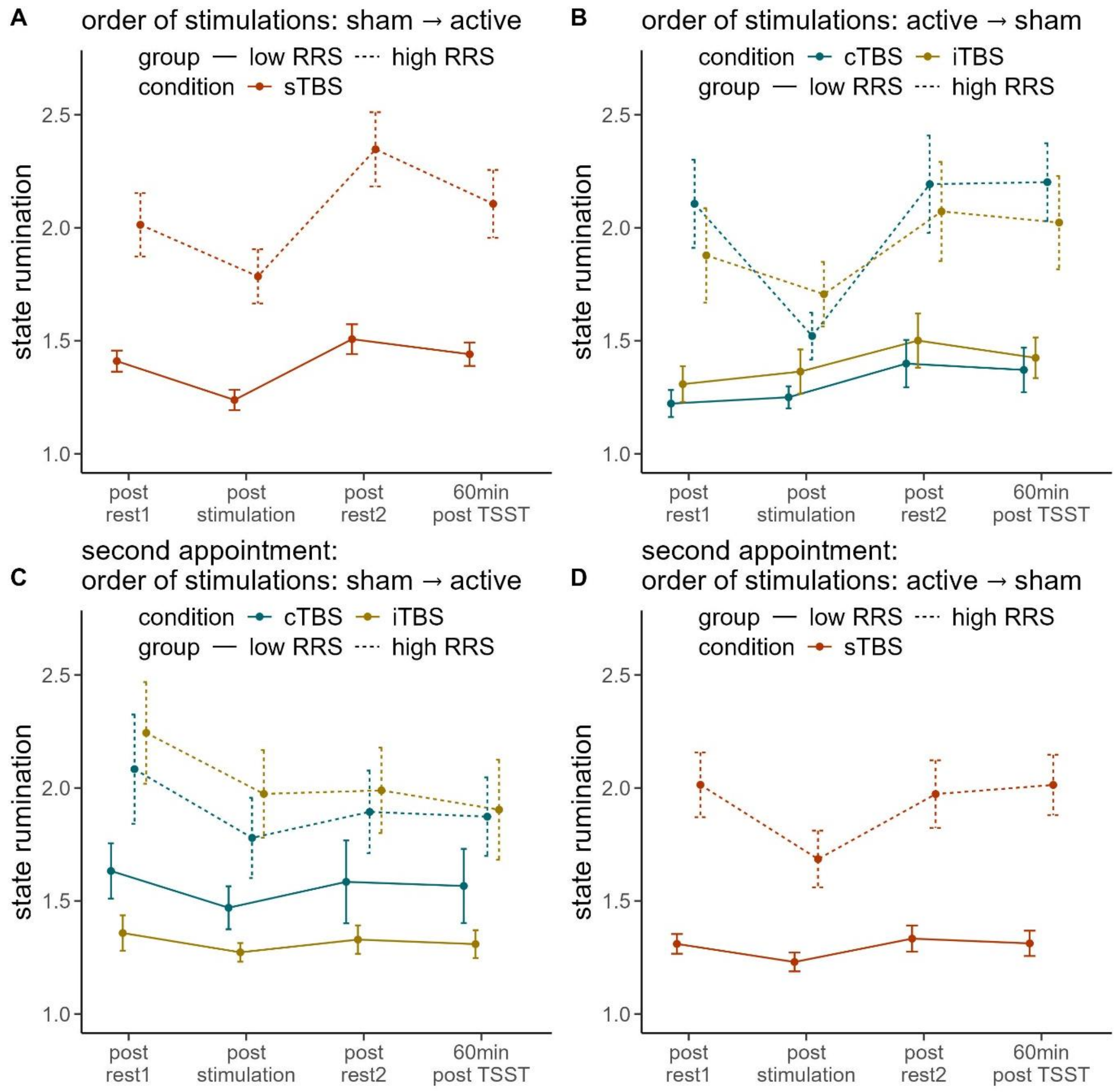

Figure S12.2. Line plot of raw data of state rumination ratings dependent on order of conditions and appointment (A = first appointment sham stimulation; B = first appointment active stimulation; C = second appointment active stimulation; D = second appointment sham stimulation). rest = resting-state measurement, TSST = Trier Social Stress Test, RRS = Ruminative Response Scale. Error bars indicate 1 standard error of the mean.

.

Negative affect raw data

Investigating the raw data using a rmANOVA dependent on the three-way interaction of time (indicating the 4 repeated assessments of the PANAS), RRS-group (low vs. high ruminators) and stimulation condition (cTBS vs. iTBS vs. sTBS) dependent on appointment, we observed a significant main effect of time (AP1: $F(2.060, 164.794) = 56.824$, $p < .001$, $\eta_p^2 = .415$; AP2: $F(2.017, 163.383) = 19.439$, $p < .001$, $\eta_p^2 = .194$). In both appointments, we observed quadratic time courses (AP1: $F(1, 80) = 64.606$, $p < .001$, $\eta_p^2 = .447$; AP2: $F(1, 81) = 19.463$, $p < .01$, $\eta_p^2 = .194$), indicating significant increases in negative affect due to the stress induction and decreases again post-stress. For both appointments, we also observed a significant main effect of RRS-group, indicating overall higher negative affect in the case of high ruminators (AP1: $F(1, 80) = 13.786$, $p < .001$, $\eta_p^2 = .147$; AP2: $F(1, 81) = 30.127$, $p < .001$, $\eta_p^2 = .271$).

Cortical oxygenation raw data ROIs

Then, we fitted a rmMANOVA using our 5 ROIs (left and right DLPFC, left and right VLPFC and SAC) dependent on the three-way interaction of time (reflecting the three tasks during which cortical oxygenation was assessed: control task 1, control task 2, and the arithmetic task of the TSST), RRS-group (low vs. high ruminators) and stimulation condition (cTBS vs. iTBS vs. sTBS) for each appointment separately. Fitting the rmMANOVA for the data of the first appointment, we only observed a significant main effect of time, $F(10, 322) = 6.150$, Pillai's trace = 0.321, $p < .001$, $\eta_p^2 = .160$. Benjamini-Hochberg corrected univariate tests indicated a significant main effect of time ($p < .05$) in case of all ROIs except for the bilateral VLPFC. Polynomial contrasts revealed linear time courses in all ROIs except for the left VLPFC where we observed a quadratic time course (left DLPFC: $F(1, 82) = 19.932$, $p < .001$, $\eta_p^2 = .196$, left VLPFC, $F(1, 82) = 6.463$, $p < .05$, $\eta_p^2 = .073$, right DLPFC, $F(1, 82) = 13.092$, $p < .001$, $\eta_p^2 = .138$ and SAC, $F(1, 82) = 44.586$, $p < .001$, $\eta_p^2 = .352$).

Benjamini-Hochberg-corrected between-subjects effects indicated a significant main effect of stimulation condition ($p < .05$) in the left DLPFC at the first appointment. This effect seemed to be driven by generally lower cortical oxygenation following sTBS.

Fitting the rmMANOVA for the data of the second appointment resulted in a significant interaction of time, condition and RRS-group, $F(20, 652) = 1.594$, Pillai's trace = 0.186, $p < .05$, $\eta_p^2 = .047$. However, univariate tests indicated no significant three-way interaction in case of no ROI, neither previous nor after correction for multiple comparisons.

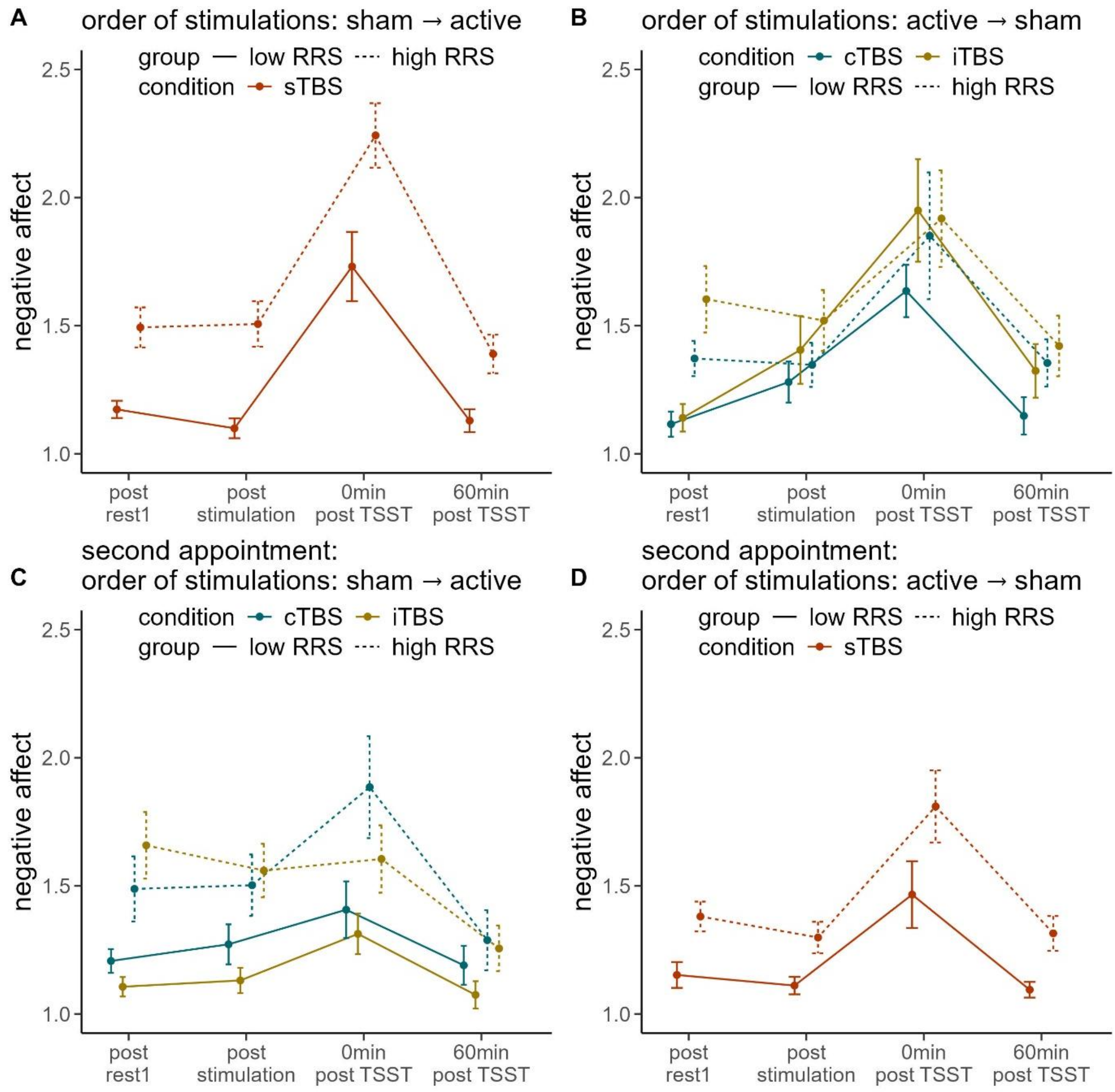

Figure S12.3. Line plot of raw data of negative affect ratings dependent on order of conditions and appointment (A = first appointment sham stimulation; B = first appointment active stimulation; C = second appointment active stimulation; D = second appointment sham stimulation). rest = resting-state measurement, TSST = Trier Social Stress Test. Error bars indicate 1 standard error of the mean.

Supplementary material S13: Positive affect contrasts

For positive affect ratings, we observed 2 multivariate outliers and 2 participants with missing data. Fitting a rmANOVA, we observed a significant main effect of time, $F(2.883, 221.963) = 4.306$, $p < .01$, $\eta_p^2 = .053$, and a significant main effect of order of stimulation conditions, $F(1, 77) = 8.738$, $p < .01$, $\eta_p^2 = .102$.

Polynomial contrasts of the main effect of time revealed a significant linear contrast, $F(1, 77) = 5.592$, $p < .05$, $\eta_p^2 = .068$. This is reflected by comparable positive ratings between active and sham stimulation previous to the stimulation and stress induction and more increasing differences afterwards. Following the TSST, we observed positive contrasts, that means higher positive affect ratings in case of the active stimulation irrespective of the order of stimulation conditions (see figure S13A and S13B).

Investigating the main effect of order of stimulation conditions, we observed overall comparable ratings between active and sham stimulation in case participants received sham stimulation first (see figure S13A) and positive contrasts (i.e. higher positive affect following active vs. sham stimulation) in case participants received active stimulation first (see figure S13B).

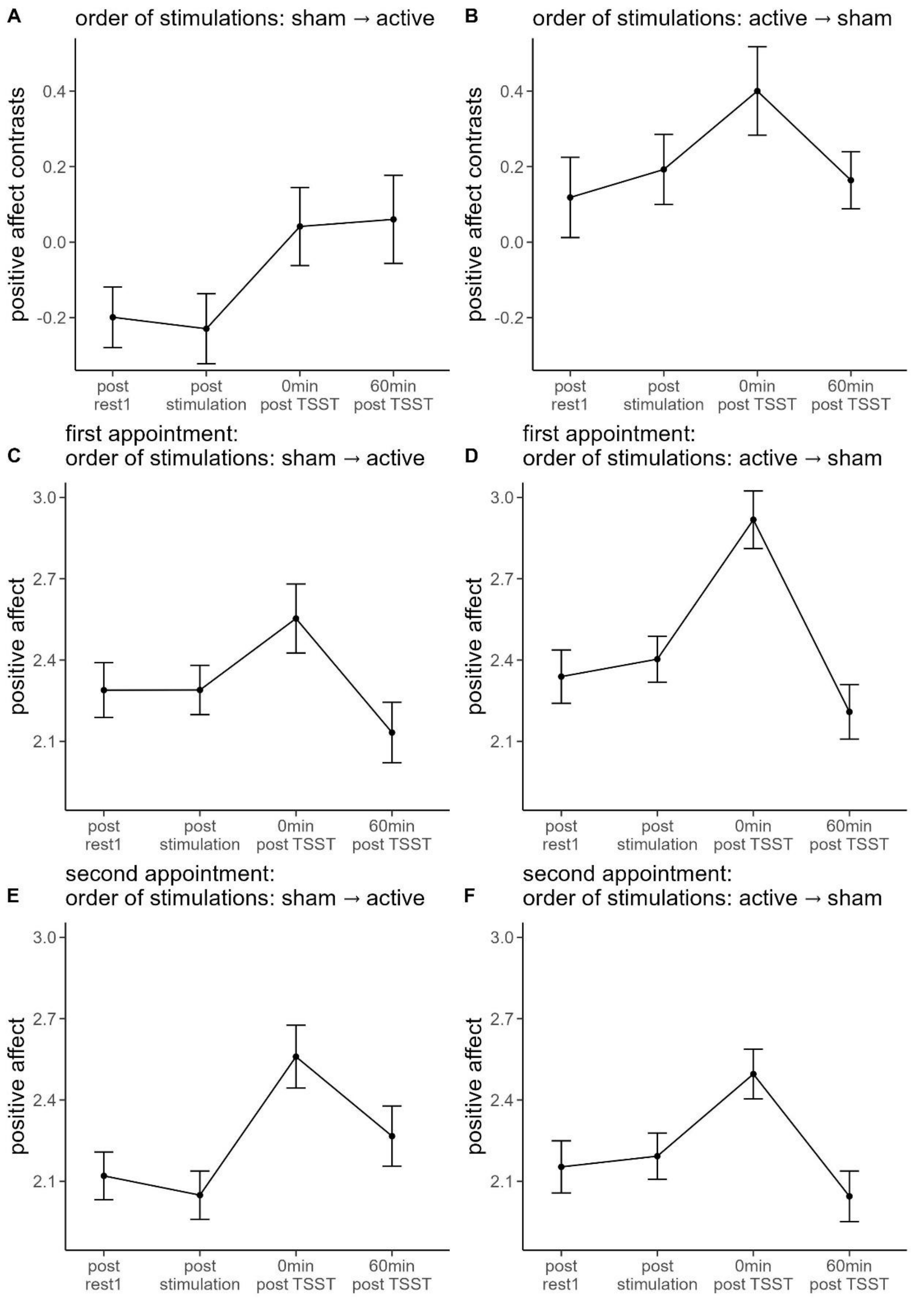

Figure S13. Line plot of the contrasts of positive affect ratings dependent on order of conditions (A = sham stimulation at the first appointment, active stimulation at the second appointment; B = active stimulation at the first appointment, sham stimulation at the second appointment) and line plot of raw data of positive affect ratings dependent on order of conditions and appointment (C = first appointment sham stimulation; D = first appointment active stimulation; E = second appointment active stimulation; F = second appointment sham stimulation). rest = resting-state measurement, TSST = Trier Social Stress Test. Error bars indicate 1 standard error of the mean.

Supplementary material S14: Number of performed calculations contrasts

No multivariate outliers ($p < .001$) were apparent for the number of performed calculations but data was missing in one participant. Fitting our rmANOVA, we observed a significant interaction of time and order of stimulation conditions, $F(1.311, 104.887) = 4.127$, $p < .05$, $\eta_p^2 = .049$, as well as a lower-order significant main effect of order of stimulation conditions, $F(1, 80) = 4.340$, $p < .05$, $\eta_p^2 = .051$.

Next, we fitted separate rmANOVAs dependent on the order of stimulation conditions. As a result, we observed a significant main effect of time, $F(1.303, 50.305) = 4.293$, $p < .05$, $\eta_p^2 = .095$, but only in case participants received sham stimulation first (see figure S14A). Polynomial contrasts revealed a quadratic time course, $F(1, 41) = 11.335$, $p < .01$, $\eta_p^2 = .217$, which is reflected by comparable number of read-out numbers (control task 1) following active and sham stimulation but positive contrasts in the case of control task 2 and the arithmetic task of the TSST (more calculations performed in case of the second appointment, i.e. active stimulation).

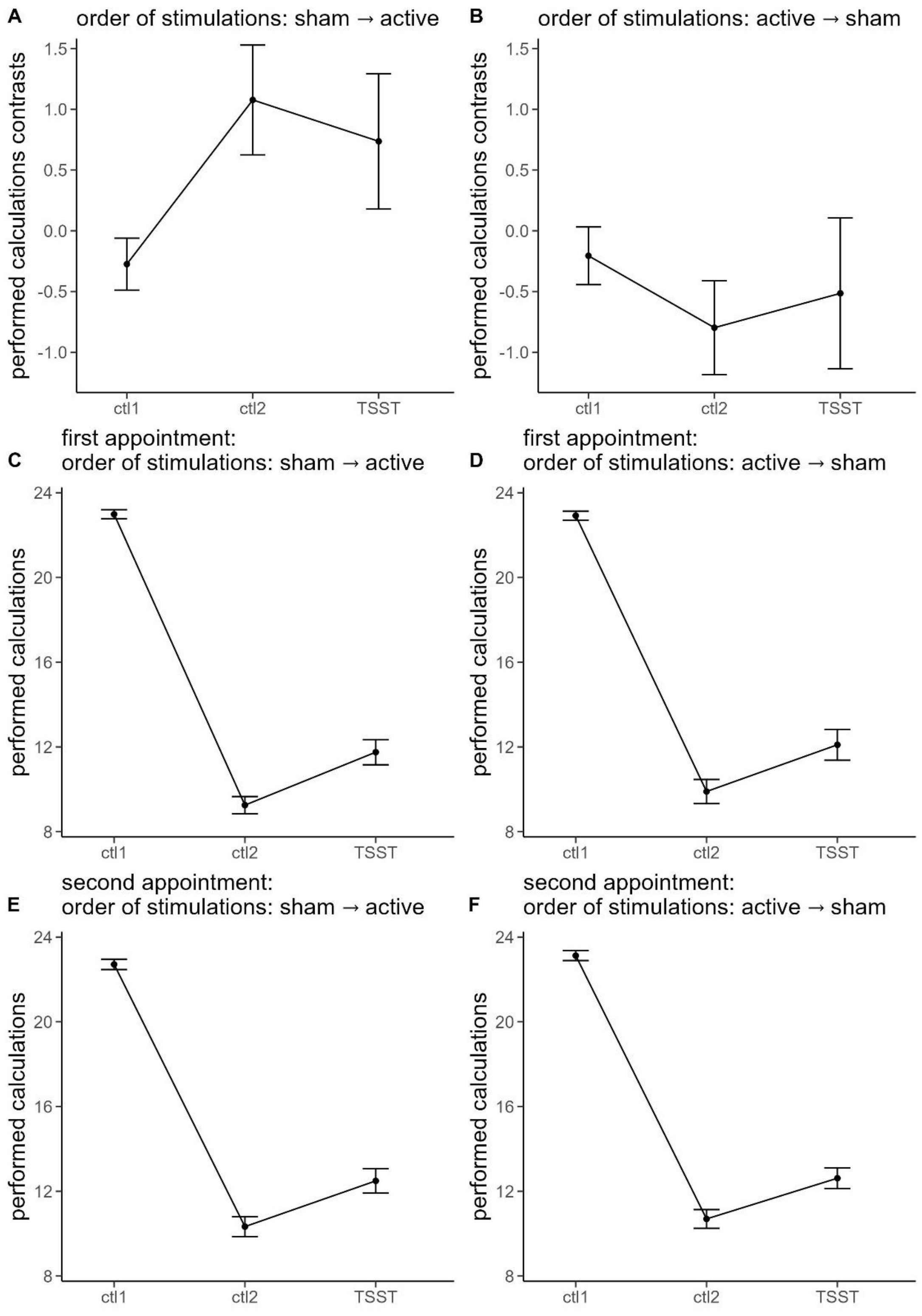

Figure S14. Line plot of the contrasts of the number of performed calculations dependent on order of conditions (A = sham stimulation at the first appointment, active stimulation at the second appointment; B = active stimulation at the first appointment, sham stimulation at the second appointment) and line plot of raw data of the number of performed calculations dependent on order of conditions and appointment (C = first appointment sham stimulation; D = first appointment active stimulation; E = second appointment active stimulation; F = second appointment sham stimulation). ctl1 = control task 1, ctl2 = control task 2, TSST = arithmetic task of the Trier Social Stress Test, Error bars indicate 1 standard error of the mean.

Supplementary material S15: Number of errors contrasts

The data of one participant was flagged as an outlier (*p* < .001) and data was missing in one participant. Fitting our rmANOVA, we observed no significant effects (all *p*'s > .100).

Supplementary material S16: Heart rate contrasts

One participant was flagged as a multivariate outlier (*p* < .001) and was therefore excluded. Data was missing in 19 participants. As a result of our rmANOVA, we observed a significant interaction of time and order of stimulation conditions, $F(4.537, 276.777) = 4.561$, $p < .001$, $\eta_p^2 = .070$, and a marginally significant lower-order main effect of time, $F(4.537, 276.777) = 2.138$, $p = .068$, $\eta_p^2 = .034$. We then fitted separate rmANOVAs dependent on the order of stimulation conditions and found a significant main effect of time but only in participants having received active stimulation first, $F(3.432, 106.395) = 4.762$, $p < .01$, $\eta_p^2 = .133$ (see figure S16B). Polynomial contrasts revealed a linear time course of the contrasts, $F(1, 31) = 8.137$, $p < .01$, $\eta_p^2 = .208$.

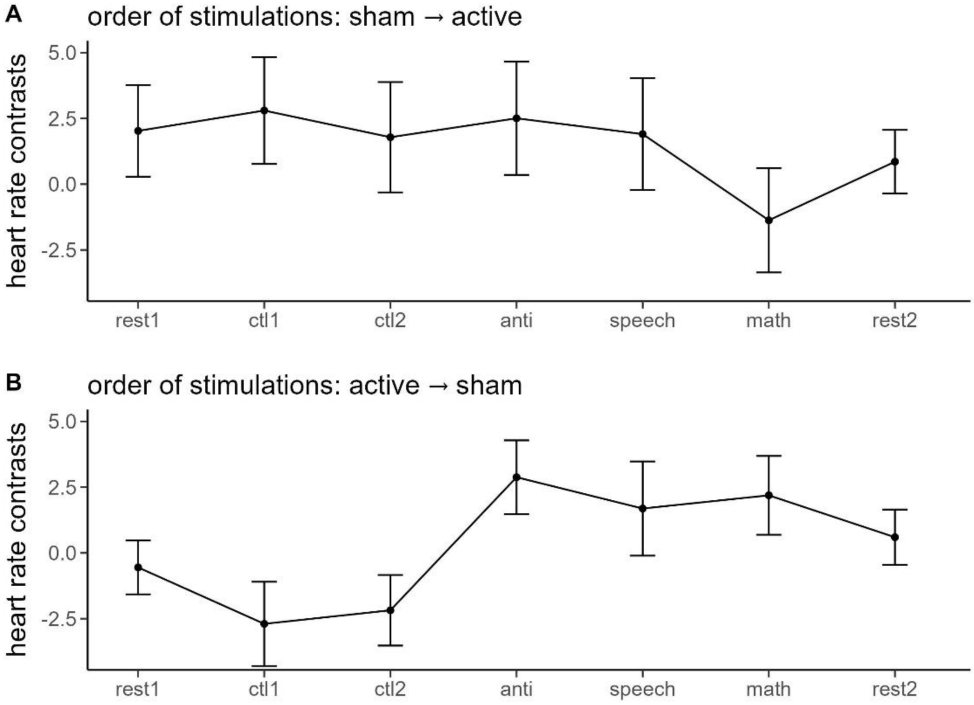

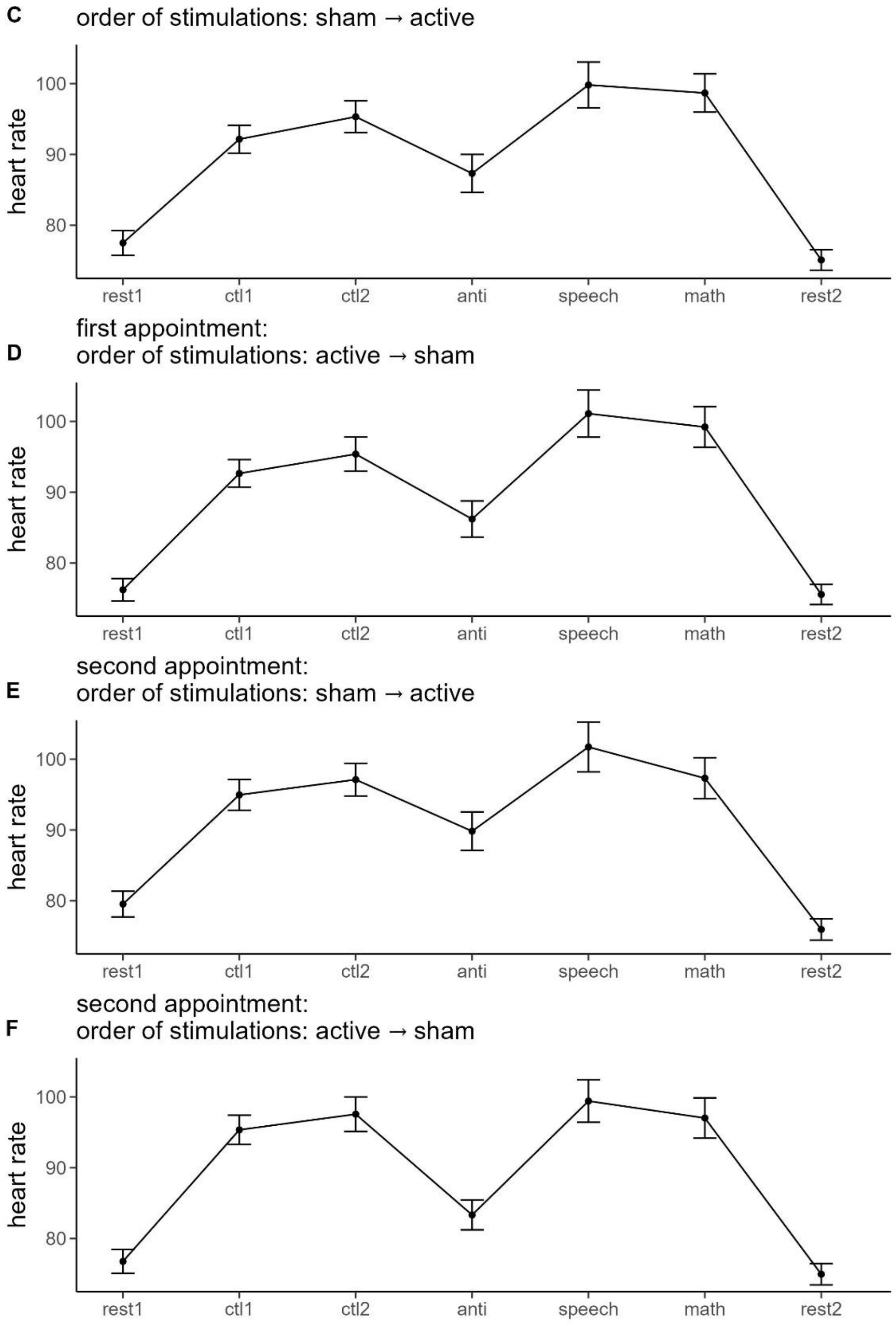

Figure S16. Line plot of the contrasts of heart rates in beats per minute (BPM) dependent on order of conditions (A = sham stimulation at the first appointment, active stimulation at the second appointment; B = active stimulation at the first appointment, sham stimulation at the second appointment) and line plot of raw data of heart rates in beats per minute (BPM) dependent on order of conditions and appointment (C = first appointment sham stimulation; D = first appointment active stimulation; E = second appointment active stimulation; F = second appointment sham stimulation). rest = resting-state measurement, ctl1 = control task 1, ctl2 = control task 2, speech = job interview of the TSST, math = arithmetic task of the TSST, TSST = Trier Social Stress Test, sTBS = sham theta burst stimulation, cTBS = continuous theta burst stimulation, iTBS = intermittent theta burst stimulation, 15 min = 15 min after the TSST. Error bars indicate 1 standard error of the mean.

Supplementary material S17: Time series of the hemodynamic responses during the arithmetic task of the TSST

Illustration of the z-standardized hemodynamic responses during the arithmetic task of the TSST in the Regions of Interest in low and high ruminators depending on the TBS-condition. A and B illustrate the raw data, while C and D illustrate the contrasts (cTBS minus sTBS and iTBS minus sTBS). The light shading marks the 40 s trial and the dark shading the 20 s pause to allow the hemodynamic response to recover. Shadings around the hemodynamic curves reflect standard errors of the mean. The baseline includes the 5 s before each trial; 0 s on the x-axis marks the beginning of the trial.

Time series of the first appointment:

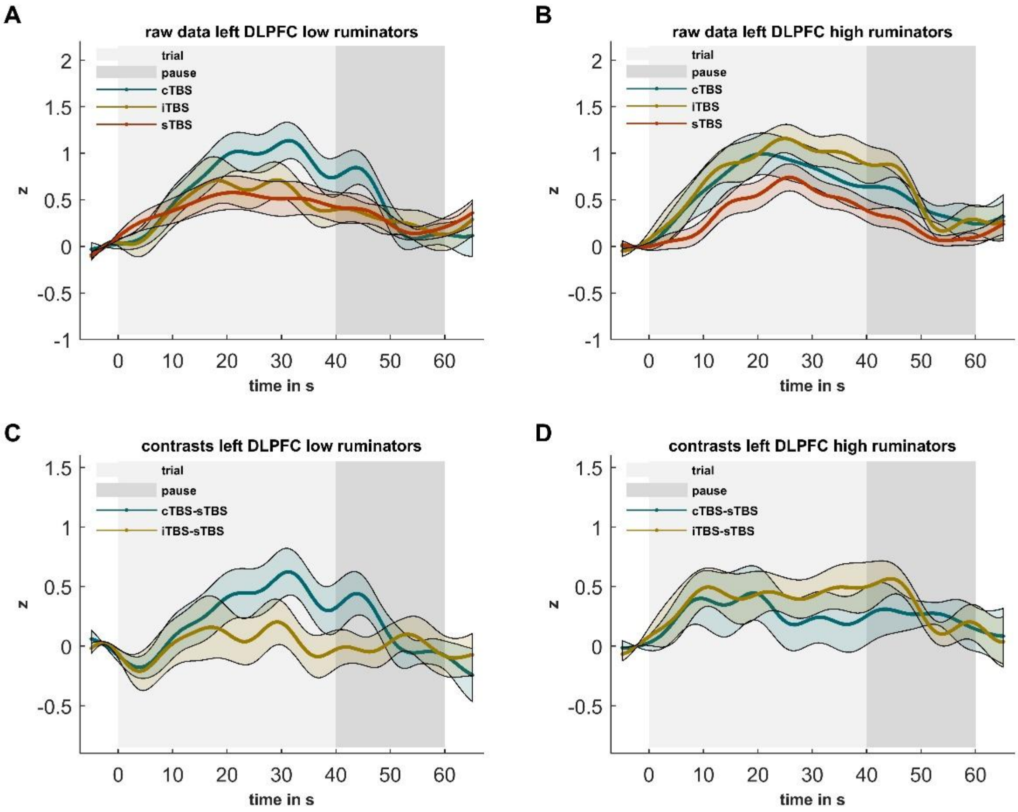

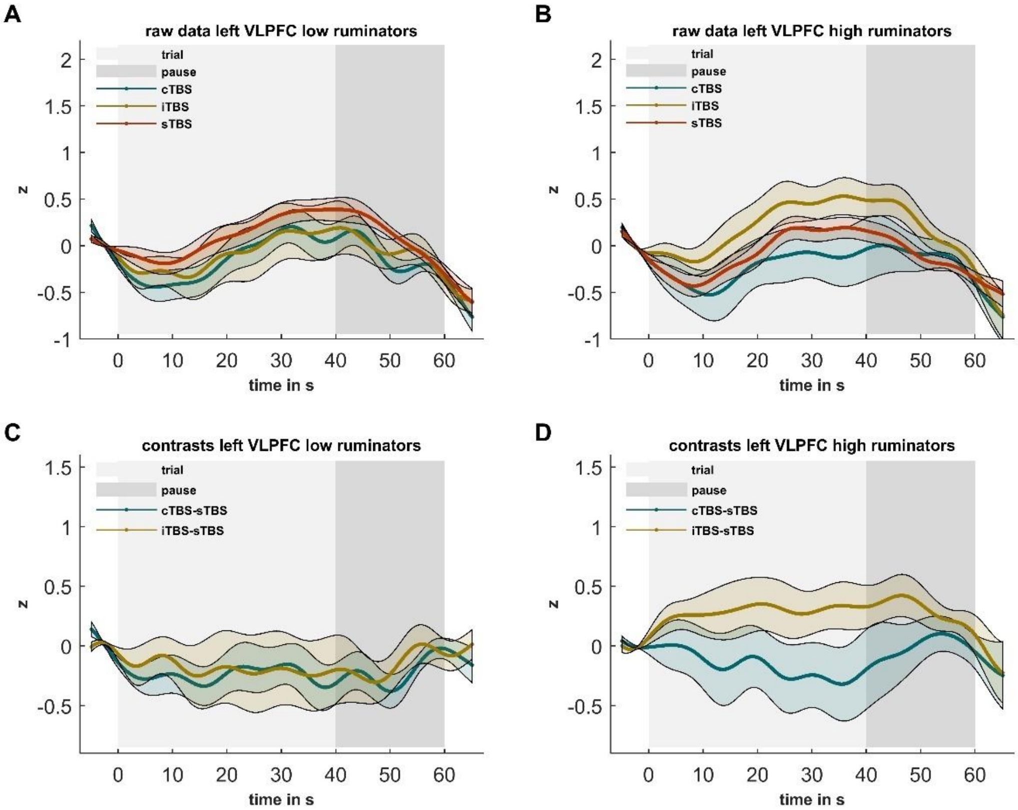
A
raw data left VLPFC low ruminators
B
raw data left VLPFC high ruminators
C
contrasts left VLPFC low ruminators
D
contrasts left VLPFC high ruminators
trial
pause
cTBS
iTBS
sTBS
cTBS-sTBS
iTBS-sTBS
z
time in s

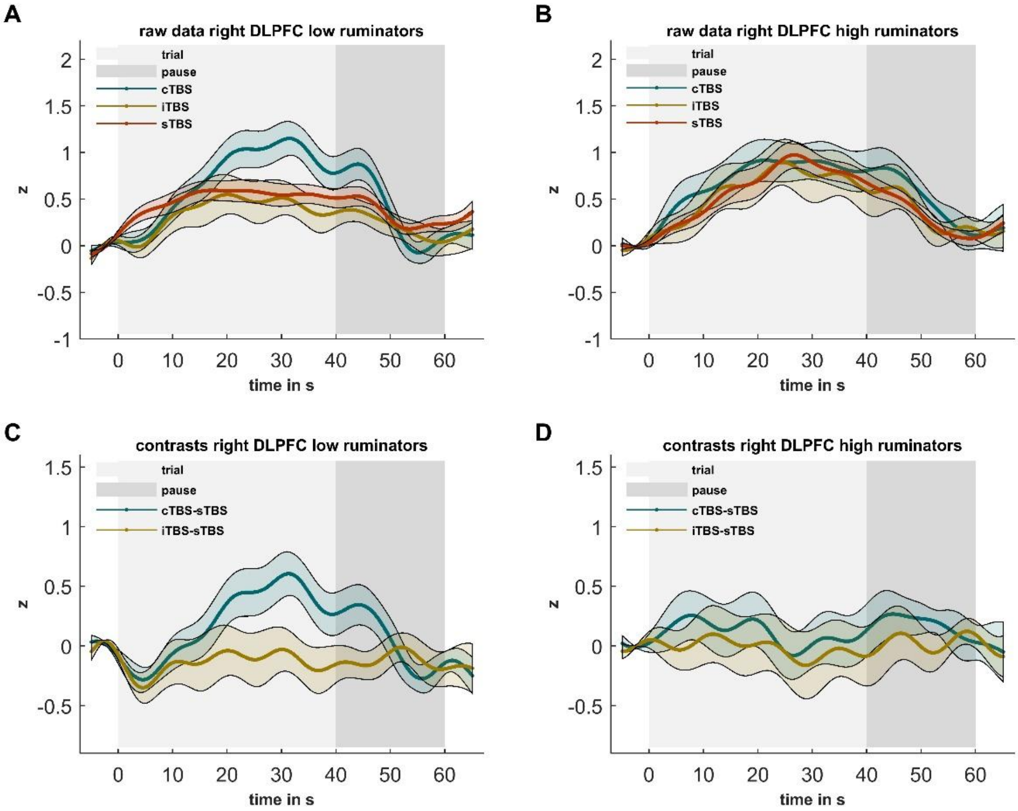
A
raw data right DLPFC low ruminators
B
raw data right DLPFC high ruminators
C
contrasts right DLPFC low ruminators
D
contrasts right DLPFC high ruminators
trial
pause
cTBS
iTBS
sTBS
cTBS-sTBS
iTBS-sTBS
z
time in s

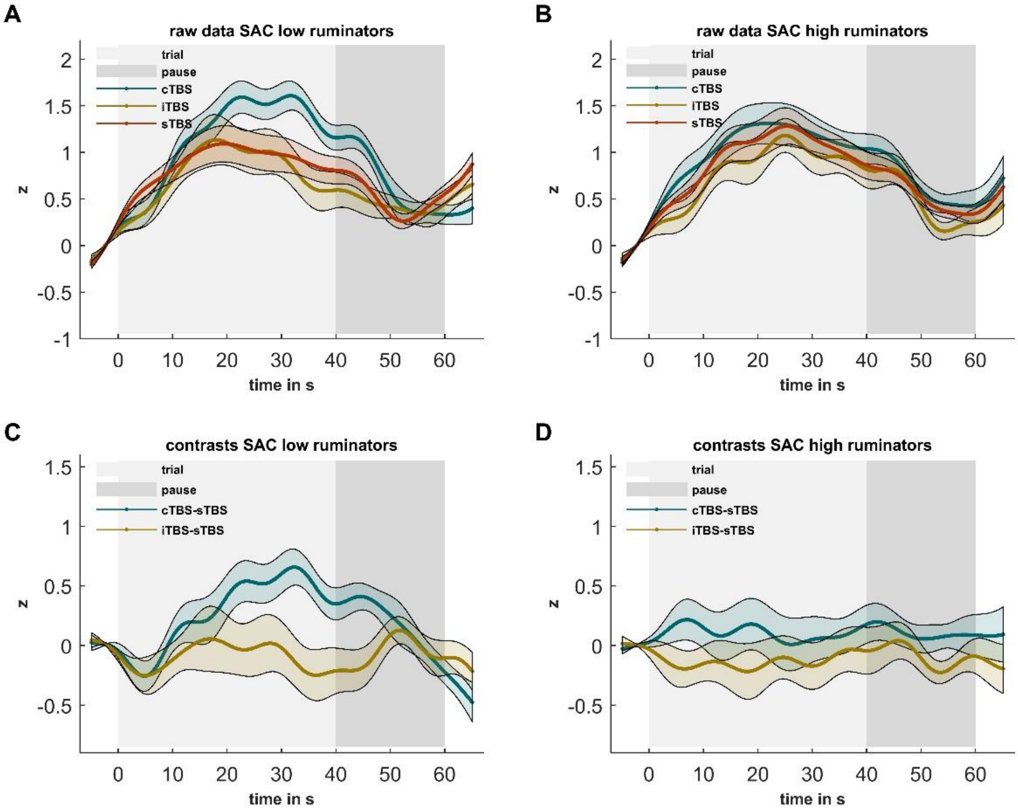

Time series of the second appointment:

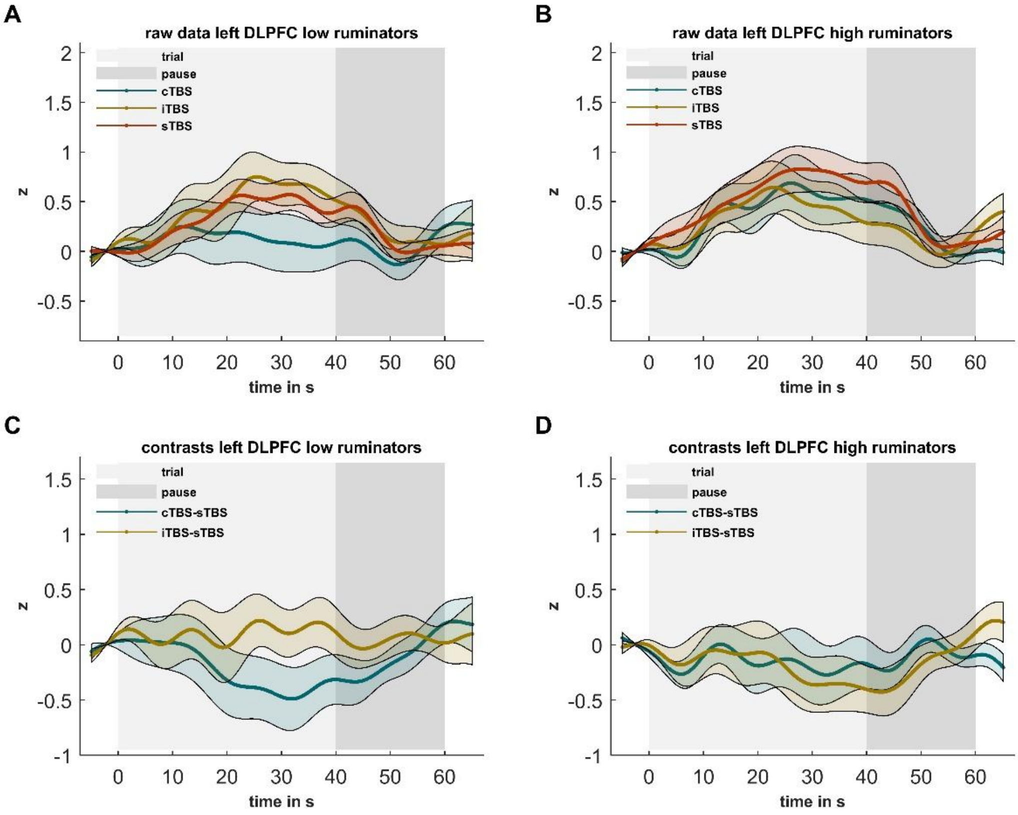

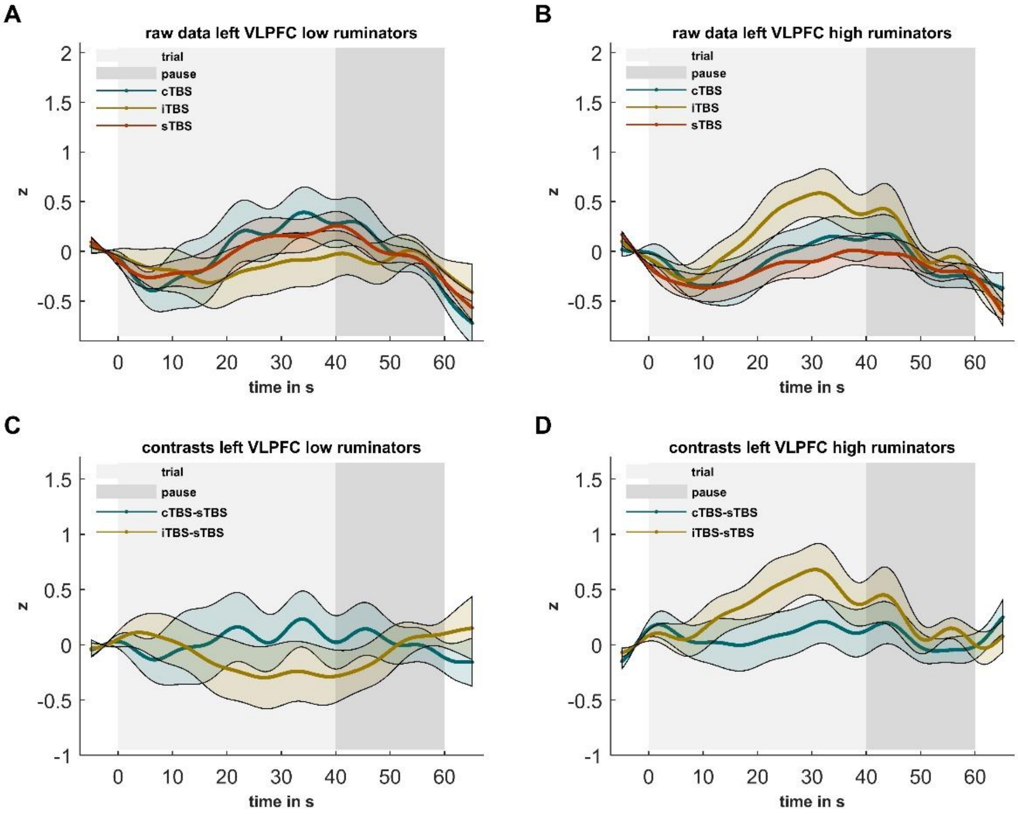
A
raw data left VLPFC low ruminators
B
raw data left VLPFC high ruminators
C
contrasts left VLPFC low ruminators
D
contrasts left VLPFC high ruminators
trial
pause
cTBS
iTBS
sTBS
cTBS-sTBS
iTBS-sTBS
z
time in s

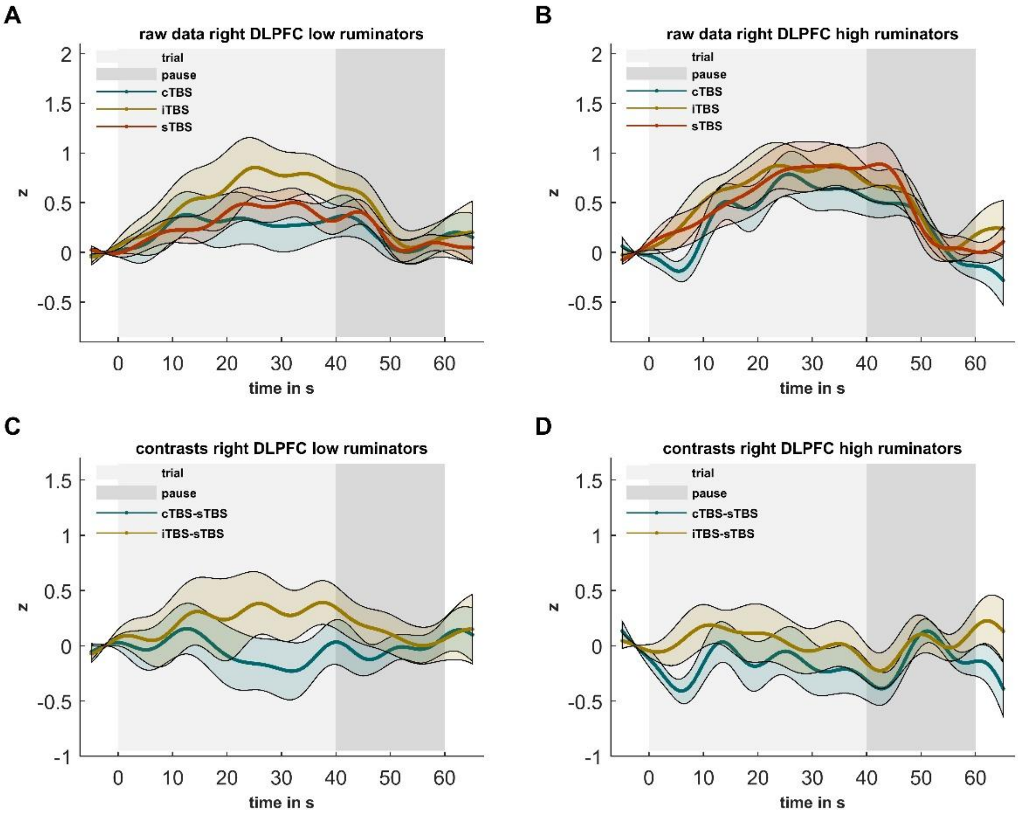
A
raw data right DLPFC low ruminators
B
raw data right DLPFC high ruminators
C
contrasts right DLPFC low ruminators
D
contrasts right DLPFC high ruminators
trial
pause
cTBS
iTBS
sTBS
cTBS-sTBS
iTBS-sTBS
z
time in s

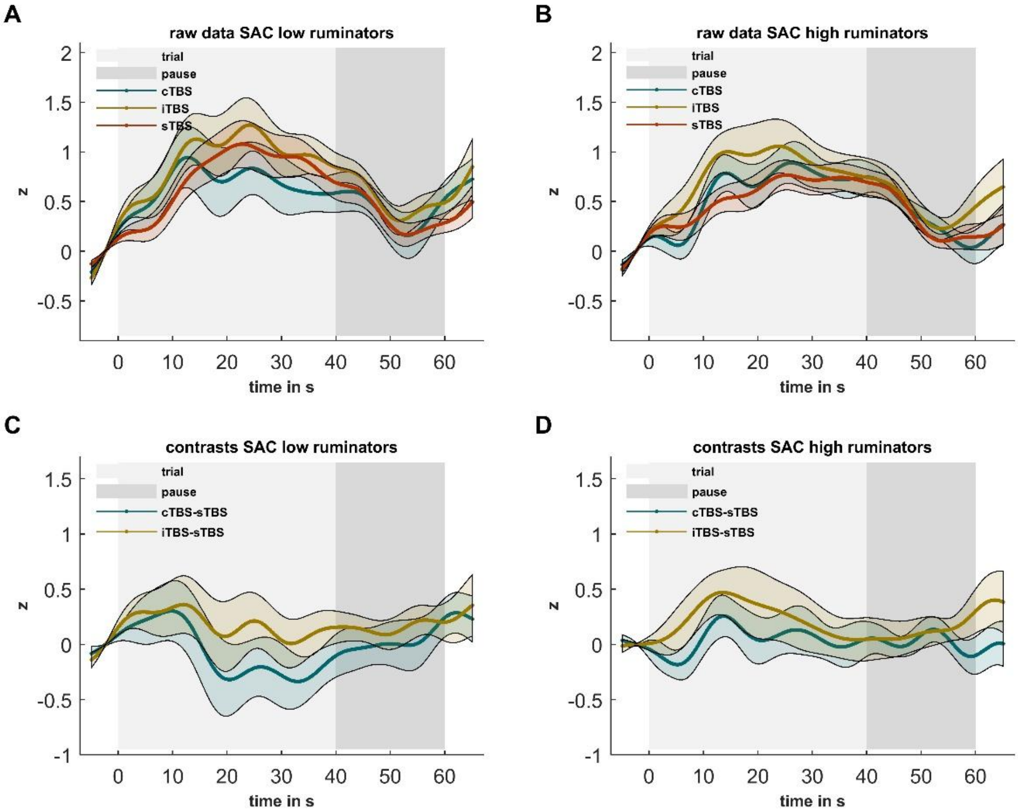

A
raw data SAC low ruminators
B
raw data SAC high ruminators
C
contrasts SAC low ruminators
D
contrasts SAC high ruminators
trial
pause
cTBS
iTBS
sTBS
cTBS-sTBS
iTBS-sTBS
z
time in s

References of the supplementary material

1. First, M., Williams, J., Karg, R. & Spitzer, R. *Structured Clinical Interview for DSM-5 Disorders, Clinical Trials Version (SCID-5-CT)*. (American Psychiatric Association, Arlington, VA, 2015).
2. De Witte, S. *et al.* The effect of neurostimulation applied to the left dorsolateral prefrontal cortex on post-stress adaptation as a function of depressive brooding. *Progress in Neuro-Psychopharmacology and Biological Psychiatry* **96**, 109687 (2020).
3. Rosenbaum, D. *et al.* Cortical hemodynamic changes during the Trier Social Stress Test: An fNIRS study. *NeuroImage* **171**, 107–115 (2018).
4. Rosenbaum, D. *et al.* Stress-related dysfunction of the right inferior frontal cortex in high ruminators: An fNIRS study. *NeuroImage: Clinical* **18**, 510–517 (2018).
5. Rosenbaum, D. *et al.* Insights from a laboratory and naturalistic investigation on stress, rumination and frontal brain functioning in MDD: An fNIRS study. *Neurobiology of stress* 100344 (2021).
6. Rosenbaum, D. *et al.* Neural correlates of stress-reactive rumination in depression–the role of childhood trauma and social anxiety. *Neurobiology of Stress* 100640 (2024).
7. Treynor, W. & Gonzalez, R. Rumination Reconsidered: A Psychometric Analysis. *Cognitive Therapy and Research* **27**, (2003).
8. Nolen-Hoeksema, S. & Morrow, J. A prospective study of depression and posttraumatic stress symptoms after a natural disaster: The 1989 Loma Prieta earthquake. *Journal of Personality and Social Psychology* **61**, 115–121 (1991).
9. Diaz, B. A. *et al.* The Amsterdam Resting-State Questionnaire reveals multiple phenotypes of resting-state cognition. *Frontiers in Human Neuroscience* **7**, (2013).
10. Ehring, T. *et al.* The Perseverative Thinking Questionnaire (PTQ): Validation of a content-independent measure of repetitive negative thinking. *Journal of Behavior Therapy and Experimental Psychiatry* **42**, 225–232 (2011).

11. Kirschbaum, C., Pirke, K.-M. & Hellhammer, D. H. The ‘Trier Social Stress Test’ – A Tool for Investigating Psychobiological Stress Responses in a Laboratory Setting. *Neuropsychobiology* **28**, 76–81 (1993).
12. Thielscher, A., Antunes, A. & Saturnino, G. B. Field modeling for transcranial magnetic stimulation: A useful tool to understand the physiological effects of TMS? in *2015 37th annual international conference of the IEEE engineering in medicine and biology society (EMBC)* 222–225 (IEEE, 2015).
13. Sassaroli, A. & Fantini, S. Comment on the modified Beer–Lambert law for scattering media. *Physics in Medicine & Biology* **49**, N255 (2004).
14. Fishburn, F. A., Ludlum, R. S., Vaidya, C. J. & Medvedev, A. V. Temporal Derivative Distribution Repair (TDDR): A motion correction method for fNIRS. *NeuroImage* **184**, 171–179 (2019).
15. Cui, X., Bray, S. & Reiss, A. L. Functional Near Infrared Spectroscopy (NIRS) signal improvement based on negative correlation between oxygenated and deoxygenated hemoglobin dynamics. *NeuroImage* **49**, 3039 (2010).
16. Okamoto, M. *et al.* Three-dimensional probabilistic anatomical cranio-cerebral correlation via the international 10–20 system oriented for transcranial functional brain mapping. *Neuroimage* **21**, 99–111 (2004).
17. Okamoto, M. & Dan, I. Automated cortical projection of head-surface locations for transcranial functional brain mapping. *Neuroimage* **26**, 18–28 (2005).
18. Singh, A. K., Okamoto, M., Dan, H., Jurcak, V. & Dan, I. Spatial registration of multichannel multi-subject fNIRS data to MNI space without MRI. *Neuroimage* **27**, 842–851 (2005).
